# An investigation of spatial signal transduction in cellular networks

**DOI:** 10.1186/1752-0509-6-83

**Published:** 2012-07-05

**Authors:** Aiman Alam-Nazki, J Krishnan

**Affiliations:** 1 Centre for Process Systems Engineering, Department of Chemical Engineering, South Kensington Campus, London, SW7 2AZ, UK; 2 Institute for Systems and Synthetic Biology, Imperial College London, South Kensington Campus, London, SW7 2AZ, UK

**Keywords:** Cellular signal processing, Spatial signalling, Signalling motifs, Global regulation, Diffusion, Non-linear dynamics, Networks

## Abstract

**Background:**

Spatial signal transduction plays a vital role in many intracellular processes such as eukaryotic chemotaxis, polarity generation and cell division. Furthermore it is being increasingly realized that the spatial dimension to signalling may play an important role in other apparently purely temporal signal transduction processes. It is increasingly being recognized that a conceptual basis for studying spatial signal transduction in signalling networks is necessary.

**Results:**

In this work we examine spatial signal transduction in a series of standard motifs/networks. These networks include coherent and incoherent feedforward, positive and negative feedback, cyclic motifs, monostable switches, bistable switches and negative feedback oscillators. In all these cases, the driving signal has spatial variation. For each network we consider two cases, one where all elements are essentially non-diffusible, and the other where one of the network elements may be highly diffusible. A careful analysis of steady state signal transduction provides many insights into the behaviour of all these modules. While in the non-diffusible case for the most part, spatial signalling reflects the temporal signalling behaviour, in the diffusible cases, we see significant differences between spatial and temporal signalling characteristics. Our results demonstrate that the presence of diffusible elements in the networks provides important constraints and capabilities for signalling.

**Conclusions:**

Our results provide a systematic basis for understanding spatial signalling in networks and the role of diffusible elements therein. This provides many insights into the signal transduction capabilities and constraints in such networks and suggests ways in which cellular signalling and information processing is organized to conform to or bypass those constraints. It also provides a framework for starting to understand the organization and regulation of spatial signal transduction in individual processes.

## Background

Cells consist of highly complex genetic and protein networks which allow them to respond to a variety of cues and make appropriate decisions. Thus much of the decision making in response to external cues, as well as the regulation and control of intracellular processes is achieved through complex, non-linear, and often sophisticated and subtle chemical signal processing [1]. A considerable body of work has focussed on the understanding of signal transduction and gene regulatory networks underlying a number of important processes. Increasingly, and especially in the last decade, modelling is integrated with experimental work.

In a number of signal transduction settings, the focus is either on steady state or temporal aspects of the signalling. However in many of these cases, spatial aspects are present: for instance proteins have to diffuse to the correct location for the appropriate signalling event to occur; there may be trafficking and interchange of signalling components between different compartments; likewise similar issues arise even in gene regulation and genetic processes. In most of the studies of such processes, spatial aspects are ignored, even if acknowledged. The non-incorporation of spatial effects in some of these processes may be reasonable given that for the available (or measurable) information on the system, a simplified description of various steps is sufficient. However, even here, it is not always clear a priori what roles spatial effects play, and what their significance might be (eg see [2-4]).

There are a number of other intracellular processes where spatial effects play a highly non-trivial and even central role and certainly cannot be neglected. Examples of these processes include the chemotactic migration of eukaryotic cells, polarity generation and maintenance in a number of cell types such as fungi and epithelial cells, cytokinesis and the propagation of intracellular waves of calcium [5-8]. It is worth pointing out that important spatial processes including localization and wave propagation occur in bacteria itself [9,10]. In all these processes the spatial aspects of the process combine with the complexity of the underlying networks and this makes them especially difficult to elucidate. In particular, it becomes necessary to ascertain and establish the spatial locations of multiple signalling components requiring appropriate spatiotemporal resolution and account for these aspects in modelling.

There has been an increasing interest in spatial signalling and an increasing awareness of the need to account for spatial effects in the signal transduction community [11,12]. Further there has been an acknowledgement that there is the need for appropriate understanding and conceptualization of various processes involved in spatial signalling [2,13].

In the modelling community there has been a history of modelling a number of specific intracellular and intercellular processes with spatial variation. In the recent past, modelling has focussed on spatial signal transduction in a number of contexts. One set of modelling efforts has dealt with modelling of networks which interpret gradients of chemical stimuli to make decisions: examples include gradient sensing in chemotaxis (eg. see [14,15]) and pheromone sensing in yeast [16]. Another set of modelling efforts has focussed on different aspects of cell polarization: this includes the dynamics of the Rho GTPase networks controlling migration [17], cell polarity involving autocrine EGFR signalling [18] and polarization of Cdc42 in budding yeast [19]; related studies have modelled basic aspects of cell polarization incorporating stochastic effects and feedback and applied this to budding yeast polarization [20]. Other studies have examined spatial effects in signalling which had been hitherto studied in purely temporal settings: these include studies of spatial effects in oscillating systems (Hes1 and p53-Mdm2) [4] and MAPK cascades [21]. Other models focus on spatial mechanisms coupling cell size and cell cycle [22] and the role of networks which provide spatial cues for cytokinesis specification [23] and self-organized microtubule assembly [24]. There is also a broader literature focussing on modelling self-organized behaviour at the cellular level (reviewed in [25]). These examples are a snapshot of some of the spatial signal transduction modelling in specific biological contexts and is by no means a complete list. These models exploit different spatial aspects of signalling elements which are embedded in intricate intracellular network interactions to explain certain phenomena in specific biological processes. What is lacking in the spatial signal transduction modelling literature, however, is a systematic analysis of how different types of network interactions can give rise to complex non-trivial spatial signal processing.

In this paper we will develop a modelling and systems framework to elucidate certain aspects of spatial signalling. Thus we will be concerned with various aspects of spatial variation on one hand, and with aspects of the signalling on the other. This informs our approach in this paper. Spatial signalling includes the natural complexity of signal transduction, with other aspects specific to spatial considerations. At the outset, while considering spatial signalling, we see that there are different ways in which spatial variation in signalling entities in a network can occur: (i) Some regulating signal or cue external to the cell or “upstream” of the network is spatially varying (ii) there is internal spatial variation of signalling entities for instance due to pre-existent asymmetries, spatial localization, compartmentalization or sequestration (iii) Self organized spatial or spatio-temporal behaviour [25]. Classic examples of self-organized behaviour include the generation of patterns through Turing mechanisms and their variants [26,27]. We of course note that depending on the signalling entities or process of interest, this distinction can differ: for instance in a process, a self-organized behaviour may result in spatial pattern formation, which combines with other signalling, and thus acts as a pre-existent or internal spatial variation for other interacting signalling processes. Further in a given process/system, different combinations of these factors might be important.

In this paper we will elucidate certain aspects of spatial signal processing in the first case, namely spatially varying signals upstream of a given network. Our approach will be generic in nature and not process and/or system specific. While elucidating specific spatial signalling processes of course needs to include specific experimental investigations appropriately complemented by modelling, we will not attempt that here. This is because of the diversity of processes, the time-consuming and iterative nature of modelling, and the fact that quite a few details of the process depend on the system under consideration. Our goal is to obtain insights into how spatial signals are transduced through different networks, which may be relevant in multiple contexts. In order to do this, we use as a basis a set of basic signal transduction networks or motifs. These networks are chosen because the dynamic behaviour they embody (eg. switch-like behaviour or oscillations) or their structure is widely observed in cell signalling settings. Noting that structure of course does not uniquely determine dynamic behaviour even qualitatively, we choose our basis set to represent essentially different kinds of signal processing which are observed. Thus the modules we will consider include coherent and incoherent feedforward, negative and positive feedback circuits (with no cooperativity), cyclic networks, monostable and bistable switches and oscillators. We then examine how these networks respond to spatially varying signals and further how the circuit response is affected by diffusion of individual elements. In this manner we develop a number of insights into how common basic signalling circuits process spatially varying signals and how diffusion may significantly affect the signal processing.

We believe that the use of simplified models is much more appropriate for the analysis we undertake here. It is worth pointing out that even in temporal signalling, while different signalling circuits vary considerably in details, there are some kinds of basic signalling patterns which occur repeatedly and the understanding of these basic elements has proven to be a good reference point for understanding additional complexities in signalling [28]. We adopt an analogous approach here. All the signalling circuits which we study include compact encapsulations of the signalling behaviour we wish to examine, while simultaneously allowing for exploration of the issues at hand. The nature of the analysis implies that it is possible to transfer such insights to more complex models with similar dynamic behaviour. We believe that such an approach will help in developing a basis for systematically understanding aspects of spatial signalling.

Our results provide non-trivial insights regarding temporal and spatial signal transduction and when particular temporal signal transduction characteristics may or may not be translated into spatial signalling characteristics. In particular, we find that having highly diffusible or global entities can either impose big constraints or create new capabilities in spatial signal transduction when compared to temporal signal transduction. For instance, such entities can strongly accentuate switching behavior in spatial signalling, or abolish the switching behaviour in spatial signals (in bistable switches). This aspect is explored in detail in multiple modules.

The paper is organized as follows. In the next section, we detail the various models which we use for our investigations. In the following section, we present a series of results on how these circuits process spatial signals and how diffusion might play a role in modulating or affecting the response. The results are based on numerical and analytical work, some of which is detailed in the Appendix. We then conclude with a synthesis of the results.

## Methods

In this section we present and discuss the models which we employ. Before proceeding to discuss the models in detail, we first discuss the goal of the work, and the logic for the choice of models.

The goal of this work is to examine certain aspects of spatial signalling driven by external or “upstream” signals through the investigation of representative network circuits, and to further elucidate in which regard the signal processing mirrors temporal signalling and where it is essentially different. Spatial signal transduction of course encompasses a wide variety of processes with all their underlying complexities, with details potentially varying significantly even from system to system. Our goal will therefore be to choose a class of representative signalling networks to investigate these issues. As has been mentioned earlier, the investigation and analysis of ubiquitously occurring signalling behaviour through basic minimal models has proven to be very useful in temporal signalling.

**Figure 1:**
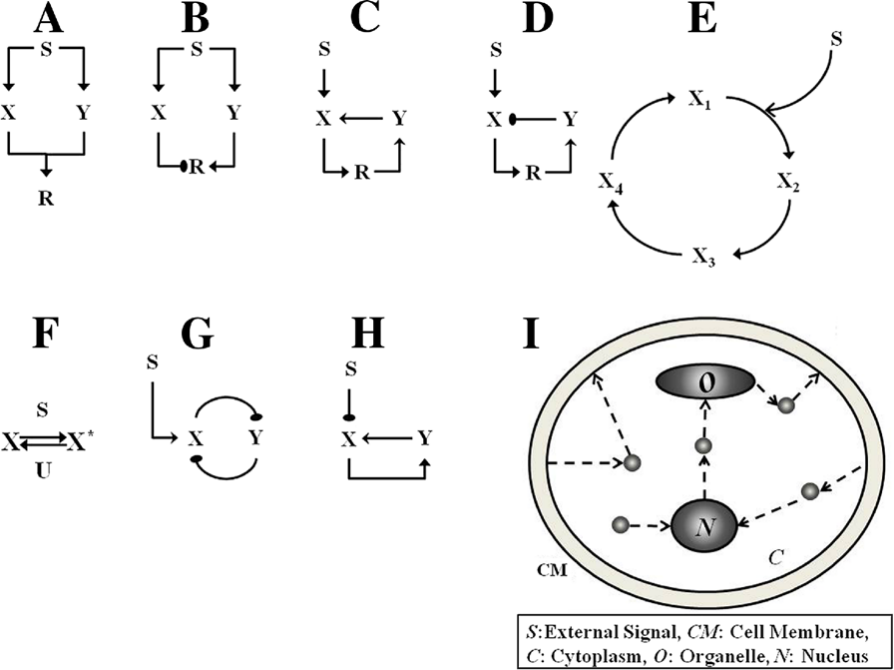
**Schematic diagrams of modules employed.** The input *S* is a spatial signal. (**A**) In the coherent feedforward module *S* positively regulates (arrows denoted by filled arrowheads) *X* and *Y * which in turn positively modulate the response *R*. (**B**) Similar to (**A**) except *X* inhibits the response element, representing one kind of incoherent feedforward circuit (see text). (**C,D**) In the positive (negative) feedback module *S* leads to the activation of the response *R* which subsequently activates (inhibits) *X* via the intermediate feedback element *Y *. (**E**) A sample cyclic reaction network is shown. *S* catalyzes (denoted by a sharp line arrowhead) the conversion of *X*_1_to *X*_2_. (**F**) The monostable switch module consists of two enzymes *S* and *U* acting close to saturation and mediating the production and degradation of *X*. (**G**) The bistable switch module consists of a double negative feedback loop. *S* regulates the production of *X* and *X* and *Y * inhibit one another. (**H**) In the transcritical bifurcation module *X* is inhibited by the signal and activates its own production via the feedback element *Y *. The oscillator module is realized from the topology in (**D**) with different non-linear interactions (see text). (**I**) A simple cross section of the cell is shown exhibiting a cell membrane (CM), cytoplasm (**C**), an organelle (O) and a nucleus (N). The small circles in the cell represent signalling molecules. Gradually graded as well as localized concentration profiles of different signalling entities naturally arise and are transduced through various complex networks which also include global coupling through diffusion.

The models which we will examine are a suite of essentially minimal models which compactly describe qualitatively different signalling patterns. These are realized through networks of a small number of components. The models are chosen because they represent signalling and causal patterns which are observed or studied in temporal signalling [28,29]. The models examined are (i) Coherent feedforward network (ii) Incoherent feedforward network (iii) Positive feedback regulation (iv) Negative feedback regulation (v) Cyclic network (vi) Monostable switches (vii) Bistable switches (viii) Oscillators (ix) Other switches (see Figure 1).

These networks are all purely temporal networks to start with, and further have a clear input-output representation. While in some cases different parameters or reactions could alternatively be modulated by an input, we will regard the designated input as the primary input, since such other modulation either has essentially similar effects or otherwise predictable effects. Since we focus on spatial signalling we will assume that these networks are spatially distributed, and thus different components can diffuse.

We will especially focus on where spatial signalling in these networks may be essentially different from temporal signalling. We will approach this as follows. For each network we will start with the case where each component is non-diffusible. We will examine the response to spatially varying signals to start with, as these are the most natural and basic ways in which spatial signal processing may occur. We will then examine the role of diffusion by examining how highly diffusible entities may distort the signal transduction in these networks, and explore what spatial analogues of these temporal signalling mechanisms may be. This will be done by considering the same classes of signals but also studying the effect of high diffusivities of network components. Taken together our studies will elucidate basic spatial processing and the significant changes introduced by highly diffusible or global components.

We now discuss the basic models which we will study. All of these are postulated on a 1-D spatial domain for simplicity. The 1-D domain could represent a membrane compartment or a slice of a cytosolic compartment or organelle. It should be noted that the essential results and insights in this paper carry through to 2-D and 3-D domains as well. Further for specificity we will assume periodic boundary conditions, although most results are equally valid for other boundary conditions such as no-flux boundary conditions. Periodic boundary conditions imply that the spatial region for modelling is akin to a circle, with the two ends identified with one another. Since none of the results in the paper actually relies on the boundaries of the spatial region under consideration, the effect of this choice of boundary condition is minimal.

In all modules the signal will be denoted by S while *R*^∗^is a key response element of interest. We will allow each element to be diffusible (discussed later). We also note that all enzymatic reactions are described by mass action kinetics, unless specifically mentioned otherwise.

In the equations below, the species with a ‘*’ superscript denotes the active form and species term without this denotes the inactive form. *D*_*j*_ is the diffusion coefficient for the species j. *θ* denotes the position of the species. In many of the circuits, the signalling model involves the interconversion between active and inactive forms a species, say *X* and *X*^∗^. In these cases, when active and inactive forms are non-diffusible, the signalling leads to conservation of total amount of *X* + *X*^∗^ =* X*_*tot*_. In such cases, we write only the equation for the rate of change of concentration of the active form *X*^∗^ in terms of *X*^∗^ and *X* and this conservation will be used to eliminate. This introduces an additional parameter *X*_*tot*_. It may be noted that if *X* and *X*^∗^are diffusible (assumed to have the same diffusivity), then it is found that *X* + *X*^∗^ satisfies a homogeneous diffusion equation, and since *X* + *X*^∗^ =* X*_*tot *_holds good initially everywhere, this continues to hold good for all time. Thus again, *X* can be written in terms of *X*^∗^ and *X*_*tot*_.

In the equations of the models 1-4 (coherent feedforward, incoherent feedforward, positive feedback, negative feedback) the kinetic constant *k*_*ij *_denotes the i species mediated conversion of species j to its active form (if the term is preceded by a positive sign) or inactive form (if the term is preceded by a negative sign), where *i *=* X* or *Y * or *R* or *S* and *j *=* X* or *Y * or *R*. *k*_*j *_and *k*_−*j *_denote the rate constants for the constitutive (basal) conversion between inactive and active forms for species j.

### Coherent feedforward module

This module involves a signal regulating a response element through two parallel pathways, denoted by X and Y (with active forms denoted by *X*^∗^and *Y*^∗^). Both *X*^∗^ and *Y*^∗^ additively regulate the production of *R*^∗^in a positive way. The equations governing the coherent feedforward module are given by

(1) $$\begin{array}{l} \frac{\partial X^{*}}{\partial t}=(k_{\text{sx}}S+k_{x})X-k_{-x}X^{*}+D_{X}\frac{\partial^{2}X^{*}}{\partial \theta^{2}}\\ \frac{\partial Y^{*}}{\partial t}=(k_{\text{sy}}S+k_{y})Y-k_{-y}Y^{*}+D_{Y}\frac{\partial^{2}Y^{*}}{\partial \theta^{2}}\;\\ \frac{\partial R^{*}}{\partial t}=(k_{\text{xr}}X^{*}+k_{\text{yr}}Y^{*})R+k_{r}R-k_{-r}R^{*}+D_{R}\frac{\partial^{2}R^{*}}{\partial \theta^{2}} \end{array}$$

In the above equations the parameters *k*_*xr *_and *k*_*yr *_were varied to modulate the strength of the associated pathways.

### Incoherent feedforward module

Here, in contrast to the first module, the signal regulates the response element through two pathways X and Y which are in opposition to one another. Thus, we have Y positively regulating the production of the response element, while X negatively regulates it. There are two ways in which this could be modelled. In one case, X inhibits the production of the response element (by for instance binding to and suppressing the enzymatic activity involved) while in the second case, X acts to enhance the deactivation/degradation of the response element. We choose the second case first, and subsequently consider the first case. The equations governing the incoherent feedforward module in the second case mentioned above (also see [30,31]) are given by

(2) $$\begin{array}{l} \frac{\partial X^{*}}{\partial t}=(k_{\text{sx}}S+k_{x})X-k_{-x}X^{*}+D_{X}\frac{\partial^{2}X^{*}}{\partial \theta^{2}}\;\\ \frac{\partial Y^{*}}{\partial t}=(k_{\text{sy}}S+k_{y})Y-k_{-y}Y^{*}+D_{Y}\frac{\partial^{2}Y^{*}}{\partial \theta^{2}}\;\\ \frac{\partial R^{*}}{\partial t}=k_{\text{yr}}Y^{*}R-k_{\text{xr}}X^{*}R^{*}+k_{r}R-k_{-r}R^{*}+D_{R}\frac{\partial^{2}R^{*}}{\partial \theta^{2}} \end{array}$$

Here X is the inhibitor and Y is the activator. In this module *k*_*sx*_,*k*_−*x*_,*k*_*xr*_,*k*_*sy*_,*k*_−*y*_ and *k*_*yr*_ are modulated to achieve different scenarios where either X or Y is regulated more strongly.

We also consider another type of incoherent feedforward module where the signal S modulates X positively and Y negatively. Both X and Y additively regulate the activation of *R*^∗^.

(3) $$\begin{array}{l} \frac{\partial X^{*}}{\partial t}=(k_{\text{sx}}S+k_{x})X-k_{-x}X^{*}+D_{X}\frac{\partial^{2}X^{*}}{\partial \theta^{2}}\\ \frac{\partial Y^{*}}{\partial t}=k_{y}Y-(k_{\text{sy}}S+k_{-y})Y^{*}+D_{Y}\frac{\partial^{2}Y^{*}}{\partial \theta^{2}}\\ \frac{\partial R^{*}}{\partial t}=(k_{\text{xr}}X^{*}+k_{\text{yr}}Y^{*})R-k_{-r}R^{*}+D_{R}\frac{\partial^{2}R^{*}}{\partial \theta^{2}} \end{array}$$

The difference in this module is in the equation for the response element, where both *X*^∗^ and *Y*^∗^ positively regulate the response element and in that the Y pathway is inhibited by the signal (for simplicity here the basal constant *k*_*r *_is assumed to be zero.

The previous two modules were feedforward modules. We now examine modules with feedback as a key element.

### Positive feedback module

The equations governing the Positive Feedback module are given by

(4) $$\begin{array}{l} \frac{\partial X^{*}}{\partial t}=(k_{\text{sx}}S+k_{x})X-k_{-x}X^{*}+k_{\text{yx}}XY^{*}+D_{X}\frac{\partial^{2}X^{*}}{\partial \theta^{2}}\\ \frac{\partial R^{*}}{\partial t}=(k_{\text{xr}}X^{*}+k_{r})R-k_{-r}R^{*}+D_{R}\frac{\partial^{2}R^{*}}{\partial \theta^{2}}\\ \frac{\partial Y^{*}}{\partial t}=(k_{\text{ry}}R^{*}+k_{y})Y-k_{-y}Y^{*}+D_{Y}\frac{\partial^{2}Y^{*}}{\partial \theta^{2}} \end{array}$$

This module describes the signal regulating an element *X*^∗^ which regulates the response *R*^∗^. Additionally *R*^∗^can positively regulate the production of the active form of X, through the production of another element Y. Y is the intermediate feedback element. Since we focus on spatial aspects and diffusion, we explicitly describe the dynamics of this intermediate feedback element. The parameter of particular interest here is the one associated with the feedback- *k*_*yx*_. Note that the positive feedback described here involves no co-operativity.

### Negative feedback module

The negative feedback module is an analogue of the positive feedback module except that the response negatively regulates the production of the upstream element *X*^∗^. Again, here, we model that by having *R*^∗^ enhancing the degradation of *X*^∗^. The equations governing the Negative Feedback module are given by

(5) $$\begin{array}{l} \frac{\partial X^{*}}{\partial t}=(k_{\text{sx}}S+k_{x})X-k_{-x}X^{*}-k_{\text{yx}}X^{*}Y^{*}+D_{X}\frac{\partial^{2}X^{*}}{\partial \theta^{2}}\\ \frac{\partial R^{*}}{\partial t}=(k_{\text{xr}}X^{*}+k_{r})R-k_{-r}R^{*}+D_{R}\frac{\partial^{2}R^{*}}{\partial \theta^{2}}\\ \frac{\partial Y^{*}}{\partial t}=(k_{\text{ry}}R^{*}+k_{y})Y-k_{-y}Y^{*}+D_{Y}\frac{\partial^{2}Y^{*}}{\partial \theta^{2}} \end{array}$$

Y is the intermediate feedback element as before. The main parameter varied here is the one associated with the feedback - *k*_*yx*_.

### Cyclic module

We consider an irreversible cyclic module, involving *n* species where the concentration of the ith species is denoted by *X*_*i*_. The reactions are irreversible and it is assumed that the signal regulates the conversion of *X*_1_ to *X*_2_, without loss of generality. We note that this cyclic module includes as a special case, the simple reversible interconversion of two species (with one of the reactions regulated by the signal), which is realized when *n *= 2. This is the simplest reversible signalling network one can consider for spatial signal processing. The equations governing the irreversible cyclic reaction network module are given by

(6) $$\begin{array}{l} \frac{\partial X_{1}}{\partial t}=k_{n}X_{n}-k_{1}X_{1}S+D_{1}\frac{\partial^{2}X_{1}}{\partial \theta^{2}}\\ \frac{\partial X_{2}}{\partial t}=k_{1}X_{1}S-k_{2}X_{2}+D_{2}\frac{\partial^{2}X_{2}}{\partial \theta^{2}}\\ \frac{\partial X_{i}}{\partial t}=k_{i-1}X_{i-1}-k_{i}X_{i}+D_{i}\frac{\partial^{2}X_{i}}{\partial \theta^{2}}(i=3,4\mathrm{..n}) \end{array}$$

In the above equation *k*_*i*_ is the rate constant associated with the conversion from *X*_*i *_to the next member in the cycle. In the case of the conversion of *X*_1_, the presence of the external signal is written explicitly, so that while all *k*_*i*_ for *i *> 1 are first order rate constants, *k*_1_ is actually a second order rate constant, with *k*_1_*S*being the corresponding first order rate constant.

The subsequent modules we examine are examples of modules which embody highly non-linear signal processing.

### Monostable switches

While monostable switches may be realized in quite different ways, for example through combination of the effects of different stages as in MAP Kinase cascades [32], we consider another case which has been studied and used widely. This is the Goldbeter-Koshland module [33], which involves a reversible reaction pair with enzymes involved in each reaction acting close to saturation. The equations governing this module are given by

(7) $$\begin{array}{l} \frac{\partial X^{*}}{\partial t}=\frac{k_{\text{sx}}\text{sx}}{K_{M1}+X}-\frac{k_{r}X^{*}U}{K_{M2}+X^{*}}+D_{X^{*}}\frac{\partial^{2}X^{*}}{\partial \theta^{2}}\\ \;\;\frac{\partial X}{\partial t}=\frac{-k_{\text{sx}}\text{sx}}{K_{M1}+X}+\frac{k_{r}X^{*}U}{K_{M2}+X^{*}}+D_{X}\frac{\partial^{2}X}{\partial \theta^{2}} \end{array}$$

In the above equations the signal *S* is the enzyme which catalyzes the production of *X*^∗^ and the enzyme *U* catalyzes its degradation. *k*_*sx *_denotes the rate constant for the signal mediated production of *X*^∗^. The Michaelis Menten kinetic constants *K*_*M*1_ and *K*_*M*2_ are associated with the enzymes S and U, respectively. *k*_*r *_is the rate constant associated with the backward reaction.

### Bistable switches

The subsequent module we consider is a very different kind of switch, which is also ubiquitously observed in cell signalling — the bistable switch. A bistable switch is typically achieved by positive feedback [34], with co-operativity/non-linearity in the feedback regulation. The positive feedback could be realized by for instance the mutual activation/positive regulation of two elements, or alternatively through the mutual inhibition of two elements. We will present the mutual inhibition case, and discuss the similarities/differences with the mutual activation module later.

The equations governing the mutual inhibition bistable switch module are given by

(8) $$\begin{array}{l} \frac{\partial X^{*}}{\partial t}=k_{0}+k_{1}S-k_{2}X^{*}-k_{21}X^{*}Y^{*}+D_{X}\frac{\partial^{2}X^{*}}{\partial \theta^{2}}\\ \frac{\partial Y^{*}}{\partial t}=\frac{-k_{3}X^{*}Y^{*}}{K_{M3}+Y^{*}}+\frac{k_{4}Y}{K_{M4}+Y}+D_{Y}\frac{\partial^{2}Y^{*}}{\partial \theta^{2}} \end{array}$$

The above model is a sample bistable circuit analyzed in [28]. In that paper the equation for *Y*^∗^has been written explicitly as a Goldbeter Koshland switch-like function, by invoking a quasi-steady state approximation. Here we have written this in the fully expanded form- using Michaelis Menten kinetics, explicitly describing the dynamics of the two species. In the above equation, *k*_0_ is the basal rate constant for the production of species *X*^∗^. *k*_1_ denotes the rate constant for the signal mediated production of *X*^∗^. *k*_2_ and *k*_4_ denote the basal inactivation and activation rate constants for species *X* and *Y *, respectively and in each case, the associated reaction is independent of the other species. *k*_21_ and *k*_3_ are the kinetic rate constants associated with the mutual inhibition of species *X*^∗^ and *Y*^∗^ respectively. The Michaelis Menten kinetic constants *K*_*M*3_ and *K*_*M*4_ are associated with the degradation and production of *Y*^∗^respectively. This module has the property of bistability, i.e. it has two stable steady state values for a particular range of the signal values and an unstable steady state as well. The fact that a substantial signal range is associated with bistability, is readily confirmed by doing bifurcation analysis of this module with the signal as a parameter. Such analysis is performed in [28].

### Oscillator module

The module we examine is one of negative feedback leading to oscillations (studied in [28]). This is an example of a module where the strong nonlinear negative feedback is capable of generating oscillations. It should be noted that there is no explicit delay in this system.

The equations governing the Negative Feedback Oscillator module are given by

(9) $$\begin{array}{l} \frac{\partial X^{*}}{\partial t}=k_{0}+k_{1}S-k_{2}X^{*}-k_{21}X^{*}Y^{*}+D_{X}\frac{\partial^{2}X^{*}}{\partial \theta^{2}}\\ \frac{\partial R^{*}}{\partial t}=\frac{k_{3}X^{*}R}{K_{M3}+R}-\frac{k_{4}R^{*}}{K_{M4}+R^{*}}+D_{R}\frac{\partial^{2}R^{*}}{\partial \theta^{2}}\\ \frac{\partial Y^{*}}{\partial t}=\frac{k_{5}R^{*}Y}{K_{M5}+Y}-\frac{k_{6}Y^{*}}{K_{M6}+Y^{*}}+D_{Y}\frac{\partial^{2}Y^{*}}{\partial \theta^{2}} \end{array}$$

In this module, the signal is relayed through the element *X*^∗^ and *R*^∗^is the response. The negative feedback pathway involves the response inhibiting *X*^∗^ via an intermediate element *Y*^∗^(rate constant *k*_21_).

In the above equations *k*_0_ is the basal rate constant for the production of species X. *k*_1_ denotes the rate constant for the signal mediated production of *X*^∗^. *k*_2_,*k*_4_ and *k*_6_ denote the basal inactivation rate constants for the species *X*^∗^,*R*^∗^and *Y*^∗^respectively. *k*_3_ and *k*_5_ denote the rate constants associated with the conversion of the species *R*^∗^ and *Y*^∗^, respectively, from their inactive to active forms (catalyzed by *X*^∗^and *R*^∗^ respectively). The Michaelis Menten kinetic constants *K*_*M*3_ and *K*_*M*4_ are associated with the production and degradation of *R*^∗^, respectively. The Michaelis Menten kinetic constants *K*_*M*5_ and *K*_*M*6_ are associated with the production and degradation of *Y*^∗^, respectively. This three-component module consists of a negative feedback and for a critical range of values of the signal (held fixed), gives rise to a sustained oscillatory response. The lower and upper limits of the signal range where oscillations occur are associated with Hopf bifurcation points and at these points the steady state response undergoes a change in stability and oscillations are generated via supercritical Hopf bifurcations.

#### Choice of parameters

We briefly comment on the choice of parameters in the modules which we employ. An advantage of using relatively simple models is that the parametric dependence of these models can be fairly easily mapped out. As discussed above, our model analysis will first consider the response of the network to representative spatial signals, where all elements are essentially non-diffusible. Then we examine the effects of diffusion. With regard to kinetic parameters we make a representative choice of kinetic parameters in such a way that these kinetic parameters represent the typical behaviour of the module. Our aim in this paper is to focus on the spatial aspects of signalling and not get bogged down in various auxiliary analysis. If in certain modules, there are different representative parameter regimes with qualitatively different behaviour, we will analyze them separately. In the case of the non-linear dynamic modules, for the basal parameter values (which have already been considered and analyzed before), bifurcation analysis in the parameter S yields robust non-trivial parameter regimes of desired behaviour (eg. bistability, oscillations). Overall our approach is to keep auxiliary parameters in the background with the understanding that they are a reasonable choice of parameters to represent the module signalling behaviour.

We also discuss the effects of diffusion. The modules have different elements all of which may diffuse. Our main goal is to find qualitative changes introduced by diffusion and strong diffusion in particular. With that in mind, when we consider the effect of diffusion, we do so one species at a time, and choose a high enough diffusion coefficient for the domain size and time scales of interest that the element may be regarded as highly diffusible. The other species will be considered to be (essentially) non-diffusible. In this manner we will obtain clear cut qualitative signatures of the effect of highly diffusible elements in signal processing. We will not attempt to study thoroughly the effects of various diffusivities over their entire range (this will be done subsequently). We mention that the global regulation introduced by such highly diffusible components could also result from components which are exchanged between compartments, one where spatial signal transduction occurs, and another essentially well-mixed compartment which exchanges material with it. The values of all parameters can be found in Additional file 1.

The results involve performing simulations in MATLAB using ode15s. The partial differential equations were discretized using finite difference equations and the results checked by doubling the discretization. As complementary work, bifurcation/sensitivity analysis of the kinetic equations was performed using MATCONT.

## Results and discussion

In this section, we present results on the analysis of the models employed. For each module, we present two sets of results. The first relates to how the modules process spatial signals such as spatially graded signals and localized signals. Both kinds of signals are repeatedly encountered and may be generically expected in intracellular signalling. In this case the elements of the network are regarded as essentially non-diffusible. We analyze this separately firstly because it is the most natural way in which spatial signalling may arise, and secondly because it forms a basis for examining what happens when individual elements may be highly diffusible. Only steady state input signals will be examined here. We subsequently build on these results to examine what happens when individual elements may be highly diffusible. Here we will primarily focus on cases where non-trivial spatial signal transduction occurs in the network.

To perform the analysis, we use a combination of numerical simulations, bifurcation and sensitivity analysis and analytical results. Taken together the results provide insights into the spatial signal transduction capabilities of such networks, and the role of high diffusivity of individual elements in altering/distorting the local temporal signal transduction of the individual temporal modules. We build on our studies to interpret our findings in terms of implications for cell signalling associated with those modules. In particular we focus on the capabilities and constraints associated with these modules, in spatial signalling, and also on situations where spatial signal processing is essentially different from temporal signalling. We discuss these at the end of each module subsection.

### Coherent Feedforward Module

In the coherent feedforward module there are two distinct pathways involving species X and Y driving the production of the response. Here we will consider the case where the pathways are of two different strengths but similar saturation capabilities, so as to avoid dealing with additional and rather tangential issues such as saturation in the current context.

**Figure 2:**
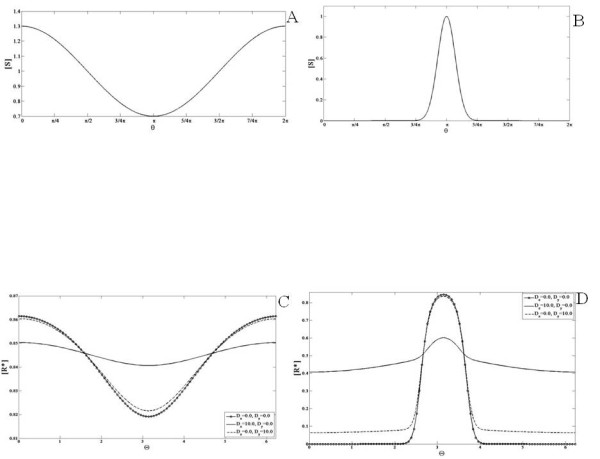
**Response of the coherent feedforward module.** An example of the (**A**) gradient signal input and the (**B**) localized signal input used in the analysis is shown (as a function of the domain postition). In this and other simulations the graded signal has a cosine variation and is of the form *S *=* a* + *bcosθ*. The localized signal is of the form *a* + *Aexp*(*x*−*x*_*o*_)/*α*. The module is subjected to a (**C**) gradient signal input and a (**D**) localized signal input and the response is shown for either when none of the elements are diffusible (solid line with circles) or when the *X* pathway (solid line) or the *Y * pathway (dashed line) is highly diffusible. The scenario where pathway *X* is stronger than pathway *Y * is shown here. In each case the response qualitatively mirrors the signal. When *X* diffuses, the spatial variation of the response is weaker than when *Y * diffuses. In both these cases, the response element exhibits weaker spatial variation when compared to the non-diffusible case.

First we will examine the case when none of the elements diffuse. When a gradient signal input (a linear gradient which amounts to a cosine signal) is applied the response essentially mirrors the input (Figure 2). When a localized signal input is applied to the module, the response is localized at the location of the input signal. Both these results are understood simply in terms of parallel feedforward effects in the coherent feedforward module.

Next we examined the case when one of the elements involved in the feedforward pathways is highly diffusible. Thus *X* and *Y * are made highly diffusible (one at a time) and the response to the aforementioned signals is again observed. The module response is qualitatively similar to the signal and has weaker spatial variation when compared to the case when none of the elements are diffusible (Figure 2). For the case illustrated in Figure 2, *k*_*xr *_>* k*_*yr*_, i.e. the mediation of the production of *R*^∗^ by species *X* is stronger relative to the *Y * mediated production of *R*^∗^. Thus, when the *X* pathway is made highly diffusible then the spatial variation of *R*^∗^ is weaker relative to the case where the *Y * pathway is highly diffusible. It is interesting to note here that in the localized response, when *X* is highly diffusible, the baseline concentration of the response is higher than the case when none of the elements diffuse. This is because the pathway involving species X conveys global information about the input signal, which results in an increase in response everywhere. It is also seen that when the stronger pathway is the highly diffusible one, this results in a higher increase in the “background” level of the output.

The above results may further be consolidated by analytical results. In the case of non-diffusible elements, basic analytical results reveal the additive effects of the pathways X and Y. Here, we will focus on the effects where one of the pathways (for specificity X) is highly diffusible. The steady state of the module is given by

(10) $$R^{*}=R_{\text{tot}}\frac{k_{\text{xr}}X^{*}+k_{\text{yr}}Y^{*}+k_{r}}{k_{\text{xr}}X^{*}+k_{\text{yr}}Y^{*}+k_{r}+k_{-r}}$$

where

(11) $$Y^{*}=Y_{\text{tot}}\frac{k_{\text{sy}}S+k_{y}}{k_{\text{sy}}S+k_{y}+k_{-y}}$$

The steady state of the element *X*^∗^satisfies the equation

(12) $$\begin{array}{ll} \;(k_{\text{sx}}S(\theta )+k_{x})(X_{\text{tot}}-X^{*})-k_{-x}X^{*}+D_{X}\frac{\partial^{2}X^{*}}{\partial \theta^{2}}=0 & \; \end{array}$$

We determine the steady state of *X*^∗^ from above, in the limit of high diffusion coefficient *D*_*X *_using a perturbation expansion, and make use of this result in subsequent modules. The complete perturbation expansion is discussed in Appendix A. We find that when X is highly diffusible, the steady state is obtained as

(13) $$X_{0}=X_{\text{tot}}\frac{k_{\text{sx}}<S>+k_{x}}{k_{\text{sx}}<S>+k_{x}+k_{-x}}$$

where <*S*> is the spatial average of the signal (see Appendix A). This simply reveals that for highly diffusible components, the steady state is essentially uniform at a level dictated by the spatial average of the signal.

From the derivation, we note that a coherent feedforward network gives rise to an output which is a combination of the direct effect of the signal through one pathway, and the global average of the signal through another pathway. It is the latter effect which results in an elevated level everywhere, even when the signal is relatively localized.

#### Biological relevance

Our analysis reveals that if the two feedforward pathway elements are non-diffusible, then the coherent feedforward pathway provides a degree of redundancy with regard to signal transduction, which is also relevant to spatial signalling. If one of the pathways is diffusible, we find that only one pathway conveys spatial information, and thus the redundancy in temporal signalling is lost in spatial signal transduction. This feature could be considered either a capability or a constraint, depending on the context.

A number of examples of the coherent feedforward module can be seen in protein signaling networks. These include the regulation of cyclin A by E2F in the cell cycle [35] and the activation of Akt by the PDGF receptor via distinct PI3K isoforms [36]. An example of the coherent feedforward network with a global element is found embedded in the signaling pathways leading to the activation of the enzyme PKC [37] through the signalling pathway associated with PLC. When PLC is activated it catalyzes the hydrolysis of PIP_2_ into Diacyl glycerol (DAG) and Inositol-3,4,5-trisphosphate (InsP_3_). DAG remains in the membrane and activates PKC and enhances its translocation to and association with the membrane. InsP_3_ diffuses into the cytoplasm and initiates the release of calcium ions in the cytosol, which in turn also activate PKC and promote its attachment to the membrane, and hence acts as the global regulator.

### Incoherent Feedforward Module

**Figure 3:**
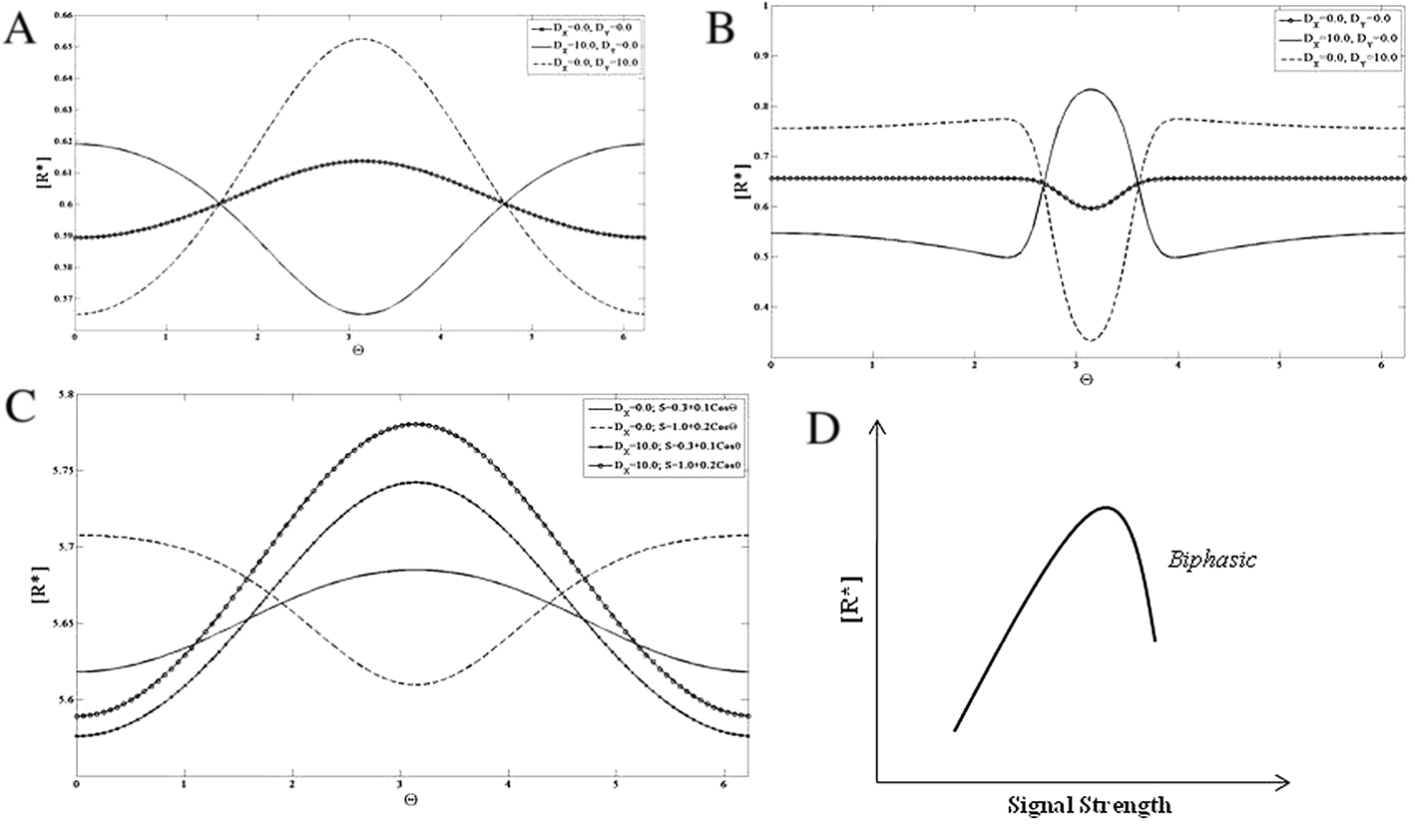
**Response of the incoherent feedforward modules.** The first incoherent feedforward module (see text) is subjected to a (**A**) gradient signal and a (**B**) localized signal input. In the case illustrated here pathway *X* (inhibitor) is stronger than pathway *Y * (activator). When none of the pathways are diffusible (solid line with circles) the response is qualitatively opposite to that of the signal. When pathway *X* is highly diffusible (solid line), the response mirrors the signal profile. On the other hand when the activator pathway is highly diffusible (dashed line) the response is qualitatively opposite to the signal. Thus, having a diffusible element allows for the response to show greater spatial contrast. (**C**) The second module (eq. 3, see text) is subjected to gradient signals. In the case illustrated here pathway X (inhibitor) is stronger than pathway Y (activator). When none of the pathways are diffusible, the response of the module switches from being qualitatively similar to the signal (solid line) to being qualitatively opposite (dashed line) to that of the signal as the mean value of the signal is increased. When pathway X is highly diffusible, the response mirrors the signal profile even if the mean value of the signal is increased (solid line with circles and solid line with x markers). Thus, the presence of a diffusible element prevents the switch in spatial biasing of the response. (**D**) A schematic of the biphasic response is shown- here the response increases and then decreases as the value of the (temporal or homogenous) signal increases.

We now examine the first type of incoherent feedforward module which has two pathways in competition with one another. This module consists of an activator *Y * and an inhibitor *X* that mediate the activation and inhibition of *R*^∗^. In our study of the incoherent feedforward module, we first modulate the strength and kinetics of these two pathways and observe the response of the module when none of the elements diffuse. In our study we will assume that the total amount of enzymes X and Y are the same. We first consider the situation, where the regulation of the X pathway is stronger than that of the Y pathway. Simulations of the module when subject to a gradual gradient reveal that the steady state concentration profile of the response indeed varies spatially and is qualitatively opposite to that of the signal (Figure 3). This is simply understood by the fact that the pathway inhibiting the response is stronger than that of the activating pathway. Similarly in response to a localized Gaussian input signal, the module exhibits a localized dip for exactly the same reason. If the activating pathway were made stronger, then the response of the module to a gradient would be qualitatively similar to that of the input, again simply reflecting the dominance of the activating pathway over the inhibiting pathway. We note in passing that if both pathways are of equal strength, then their effects exactly cancel out, resulting in a spatially homogeneous steady state output even for spatially varying input.

Next, we examined the case where one of the species was highly diffusible (see Figure 3). When the inhibitor is highly diffusible, the response of the module when subject to a gradual gradient mirrors the signal. This is irrespective of which of the two pathways has stronger kinetic regulation by the signal. This is understood by noting that only the activating pathway is conveying spatial information. There is a further difference in comparison to the non-diffusible case considered above. When the signal range is not too high (so that both pathways are far from saturation), the response has the feature that a spatially homogeneous signal leads to a steady state which is very insensitive to the level of the signal. Thus the module exhibits adaptive behaviour in a homogeneous stimulus (this is true irrespective of the diffusivity of the species). Therefore this module has the characteristic of providing a gradient response, while exhibiting adaptive behaviour in homogeneous stimuli. This means that the gradient response essentially varies about a fixed mean value (at least when the signal is not high). Thus in this case the module shows similar behaviour to the so-called Local Excitation Global Inhibition module [38]. More importantly, having a diffusible entity provides a clear contrast between temporal and spatial signalling characteristics: an adaptive response is seen in spatially homogeneous (i.e. temporal) signals, but not in spatial gradients.

If the activator is diffusible, then the module exhibits a response in a gradual gradient, which is opposite to the input simply because spatial gradient information is conveyed only through the inhibitor. This is a module which also exhibits a gradient response along with adaptive behaviour in homogeneous stimuli, with the difference that the gradient response is counter-aligned with the input.

The above results can be understood analytically. We focus on the case where one of the elements is diffusible. If the inhibitor is highly diffusible, the steady states for activator and inhibitor may be written as

(14) $$\begin{array}{l} Y^{*}=Y_{\text{tot}}\frac{k_{\text{sy}}S+k_{y}}{k_{\text{sy}}S+k_{y}+k_{-y}}\\ X^{*}=X_{\text{tot}}\frac{k_{\text{sx}}<S>+k_{x}}{k_{\text{sx}}<S>+k_{x}+k_{-x}}\\ R^{*}=R_{\text{tot}}\frac{k_{r}+k_{\text{yr}}Y^{*}}{k_{r}+k_{\text{yr}}Y^{*}+k_{-r}+k_{\text{xr}}X^{*}} \end{array}$$

In the above, since the inhibitor X is highly diffusible, the steady state is spatially uniform and depends only on the global average of the signal. This result is arrived at using a perturbation analysis exactly as considered in the previous subsection. Now if the basal rate constants *k*_*r*_,*k*_−*r*_ are small in this equation and neglected, we immediately see that the response only depends on the ratio *Y*^∗^/*X*^∗^. Now looking at the expressions for *X*^∗^and *Y*^∗^, if the forward basal rate constants *k*_*x*_,*k*_*y *_are neglected and it is further assumed that the signal strength is in a range where these pathways are far from saturation, then we have *Y*^∗^ =* k*_*sy*_*S*/*k*_−*y*_ and *X*^∗^ =* k*_*sx*_<*S*>/*k*_−*x*_. We immediately see that in a spatially homogeneous signal *Y*^∗^/*X*^∗^is independent of the signal, demonstrating the adaptation property of the response. In this case the module exhibits a co-aligned gradient response in a gradual gradient, while exhibiting an adaptive response in homogeneous stimuli. The case where the activator is diffusible is analyzed in an exactly analogous manner.

Finally we briefly examine the second type of incoherent feedforward module (see Figure 3).

An analysis of such an incoherent feedforward module reveals that for wide ranges of parameters, the behaviour is as follows: the response of the module to a spatially homogeneous stimulus is biphasic [39]. This has the following implications for spatial signalling: if the network (with all elements non-diffusible) is subject to a gradient, then the response can completely switch from being co-aligned to being counter-aligned (or vice versa) as the mean value of the signal is changed.

Next we consider the case where one of the elements in the network is highly diffusible. If the dominant pathway X is highly diffusible, then the spatial information from the signal will only be conveyed through the non-diffusible pathway- Y. Thus the response will be counter-aligned to the signal and this continues to hold good even when the mean value is changed. Thus having one pathway being diffusible, completely abolishes the possibility of switching in gradient response. This again underscores the difference between steady state temporal and spatial responses, and demonstrates how having a diffusible element acts as a constraint (if a reversal in response is desired, as has been suggested in migratory responses) or a capability (preventing a reversal in spatial response, inspite of a biphasic steady state response to temporal stimuli).

#### Biological relevance

We have just seen how having global entities in incoherent feedforward networks can prevent reversal of responses as the mean value of the driving signal is increased, and that it can also prevent adaptation in spatially varying signals. The incoherent feedforward signalling network is observed or implicated in multiple biological processes, including axon growth cone turning (involving pathways controlled by cAMP and cGMP) and hormesis or the biphasic dose response of fibroblasts to chemical agents [40,41]. Incoherent feedforward loops with diffusible entities have been used to model the PI3K and PTEN pathways underlying gradient sensing in Dictyostelium chemotaxis [14].

### Positive Feedback Module

**Figure 4:**
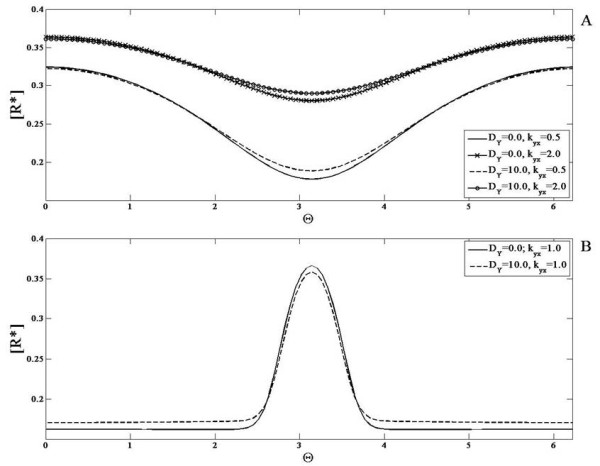
**Response of the positive feedback module.** This module is simulated with a (**A**) gradient signal and a (**B**) localized signal input and the effect of the feedback strength is analyzed for non-diffusible species and a highly diffusible feedback element. (**A**) *R*^∗^is shown for (non-diffusible case) *k*_*yx *_= 0.5 (solid line) and 2.0 (solid line with x markers) and (diffusible case) *k*_*yx *_= 0.5 (dashed line) and 2.0 (solid line with circles). (**B**) *R*^∗^is shown for (the non-diffusible case) *k*_*yx *_= 1.0 (solid line) and (the diffusible case) *k*_*yx *_= 1.0 (dashed line). The response qualitatively mirrors the signal. As the feedback constant *k*_*yx *_increases, the total concentration of the response increases everywhere. When the feedback element is highly diffusible, the spatial profile of *R*^∗^shows weaker spatial variation.

We now examine the positive feedback module. Note that the element involved in the positive feedback regulation is Y. Of particular interest in this case, will be the effect of the strength of the positive feedback. In this module, if all elements are non-diffusible, when a gradient signal input is applied, the response of the module *R*^∗^ is also a gradient co-aligned with the signal (Figure 4). Next, we observe the change in the steady state concentration profile of the response when the feedback constant *k*_*yx *_is varied, keeping the input signal fixed. As this parameter is increased, the concentration of the response is elevated at every spatial location. We then applied a localized signal input and observed the response of the module. The response obtained is localized as well.

Next, we analyzed the response of the module when the intermediate feedback element, *Y *, is highly diffusible. When the input to the module is a gradient signal, the response is a gradient signal which is co-aligned with the input signal (Figure 4) which when compared to the response in the previous analysis has a more spread out profile. Similarly, when the feedback constant *k*_*yx *_was increased, the concentration of the response everywhere increased and the resulting spatial profiles are more spread out relative to the case when the feedback element does not diffuse. Similar observations hold for the localized signal input.

These results can be complemented by analytical results. In the non-diffusible case, the steady state of the response is given by

(15) $$\begin{array}{l} R^{*}=R_{\text{tot}}\frac{k_{\text{xr}}X^{*}+k_{r}}{k_{\text{xr}}X^{*}+k_{r}+k_{-r}}\\ Y^{*}=Y_{\text{tot}}\frac{k_{\text{ry}}R^{*}+k_{y}}{k_{\text{ry}}R^{*}+k_{y}+k_{-y}}\\ X^{*}=X_{\text{tot}}\frac{k_{\text{sx}}S+k_{x}+k_{\text{yx}}Y^{*}}{k_{\text{sx}}S+k_{x}+k_{\text{yx}}Y^{*}+k_{-x}} \end{array}$$

The solution of these three equations can be achieved by eliminating variables resulting in a messy quadratic equation for *R*^∗^which has a single positive root. The effect of the feedback strength can be understood from the above equations itself.

Now if we consider the case where Y is highly diffusible, then in this case, we see that the spatial profile of Y is uniform, and by similar considerations as previous sections, we see that

(16) $$\begin{array}{l} R^{*}=R_{\text{tot}}\frac{k_{\text{xr}}X^{*}+k_{r}}{k_{\text{xr}}X^{*}+k_{r}+k_{-r}}\\ Y^{*}=Y_{\text{tot}}\frac{k_{\text{ry}}<R^{*}>+k_{y}}{k_{\text{ry}}<R^{*}>+k_{y}+k_{-y}}\\ X^{*}=X_{\text{tot}}\frac{k_{\text{sx}}S+k_{x}+k_{\text{yx}}Y^{*}}{k_{\text{sx}}S+k_{x}+k_{\text{yx}}Y^{*}+k_{-x}} \end{array}$$

What we see is that *Y*^∗^is uniform and acts as a positive feedback mediator. Thus this circuit is an example of a global positive feedback circuit, with the feedback element being uniform and spread out everywhere. It is precisely this which leads to an elevation in response everywhere.

The solutions of the above equation are more transparent for certain special cases. If we assume that all pathways are far from saturation and that basal parameters *k*_*r *_=* k*_*y *_=* k*_*x *_= 0, then in the case where all elements are non-diffusible, we have the steady state response given by (see Appendix B)

(17) $$R^{*}=\frac{\alpha S}{1-\beta }$$

*α *=* k*_*xr*_*k*_*sx*_*X*_*tot*_*R*_*tot*_/(*k*_−*r*_*k*_−*x*_) and *β *=* X*_*tot*_*Y*_*tot*_*R*_*tot*_*k*_*xr*_*k*_*yx*_*k*_*ry*_/(*k*_−*r*_*k*_−*x*_*k*_−*y*_). Note that the feedback constant is contained in the parameter *β*. In the case where the feedback element is diffusible, we have the steady state response given by (see Appendix B)

(18) $$R^{*}=\alpha S+\frac{\beta }{\left(1-\beta \right)}\alpha <S>$$

We immediately see the effect of the global feedback in the second term. Further in contrast to the non-diffusible case we see that the global feedback leads to an elevation of the response everywhere, but actually reduces the amplitude of the variation and thus weakens the spatial variation of the response.

It is also worth pointing out that in this module, if the response element was the highly diffusible element, then it would not exhibit any spatial variation at steady state. Likewise if the element X was highly diffusible, then again the response would again exhibit no spatial variation at steady state.

#### Biological relevance

Having a global element mediating the positive feedback acts to actually weaken the spatial contrast in the response. Positive feedback interactions are ubiquitously observed in signaling networks, including in different classes of MAPK cascades [42]. Signaling associated with calcium ions showcase examples of positive feedback networks with a global feedback interaction: PLC leads to the generation of IP_3_ in the cyotosol which via calcium ions acts to further activate IP_3_[43]. A simple positive feedback circuit combined with stochastic effects has been used to model polarization for budding yeast [20].

### Negative feedback module

**Figure 5:**
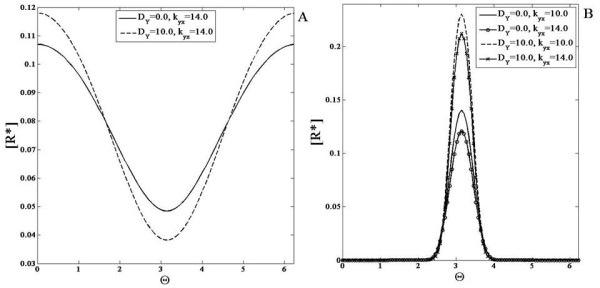
**ComparisonResponse of the negative feedback module.** This module is simulated with a (**A**) gradient signal and a (**B**) localized signal input. The response of the module when the feedback strength is varied is shown for when the feedback element *Y * is non-diffusible and highly diffusible. (**A**) *R*^∗^is shown for (non-diffusible case) *k*_*yx *_= 14.0 (solid line) and (diffusible case) *k*_*yx *_= 14.0 (dashed line). (**B**) *R*^∗^is shown for (the non-diffusible case) *k*_*yx *_= 10.0 (solid line) and 14.0 (solid line with circles) and (the diffusible case) *k*_*yx *_= 10.0 (dashed line) and 14.0 (solid line with x markers). The response exhibits a profile qualitatively similar to the signal but the concentration everywhere is reduced due to the negative feedback. As the feedback constant *k*_*yx *_increases, the total concentration of the response decreases everywhere. The spatial profile of *R*^∗^in the diffusible case shows stronger spatial variation than the corresponding non-diffusible case.

We first consider the case when none of the elements in the module are diffusible. A gradient signal input is applied to the module and the response obtained has a similar variation (Figure 5). Next, the feedback constant *k*_*yx*_ is varied and as it increases, the total concentration of the response decreases everywhere. This is due to the property of the negative feedback present in this module. A localized signal was also applied to the module and similar observations hold.

The negative feedback module is capable of exhibiting the property of adaptation - i.e. when a (homogeneous) step signal is applied to the module, a transient increase is observed in the response before it is reset at or close to its original value [44]. In the case of this simple negative feedback module, an imperfect adaptive response is observed, where the response does not decrease to its original value, but to a value a little higher (Figure 5). This is an example of an underadaptive response.

We then consider the case that the intermediate feedback element Y is highly diffusible. When a gradient signal input is applied to this module, the observed behaviour is counter-intuitive and interesting. The response exhibits greater spatial contrast than the response when Y does not diffuse (Figure 5). This is due to the global negative feedback. As the feedback constant *k*_*yx *_is increased, the concentration of the response everywhere decreases. Similar insights hold for the case when the signal is localized.

This may be understood as follows. A greater contrast in the diffusible case occurs because the inhibiting effect is global, acting in concert with a spatially varying activation. This module with global negative feedback is in some respects a feedback analogue of an incoherent feedforward module with a diffusible inhibitor. In the latter case, a gradient response is seen along with adaptation in homogeneous stimuli. In this module too, a partial adaptive response is observed, with the global negative feedback (the analogue of a diffusible inhibitor in an incoherent feedforward pathway) enhancing the spatial contrast.

The above results can be understood analytically. First considering the case that Y is non-diffusible, we see that the steady state is given by

(19) $$\begin{array}{l} R^{*}=R_{\text{tot}}\frac{k_{\text{xr}}X^{*}+k_{r}}{k_{\text{xr}}X^{*}+k_{r}+k_{-r}}\\ Y^{*}=Y_{\text{tot}}\frac{k_{\text{ry}}R^{*}+k_{y}}{k_{\text{ry}}R^{*}+k_{y}+k_{-y}}\\ X^{*}=X_{\text{tot}}\frac{k_{\text{sx}}S+k_{x}}{k_{\text{sx}}S+k_{x}+k_{\text{yx}}Y^{*}+k_{-x}} \end{array}$$

When Y is highly diffusible, the above steady state involves only one change which reflects the fact that *Y*^∗^ exhibits no spatial variation and depends on the average of *R*^∗^. Thus we have

(20) $$\begin{array}{l} R^{*}=R_{\text{tot}}\frac{k_{\text{xr}}X^{*}+k_{r}}{k_{\text{xr}}X^{*}+k_{r}+k_{-r}}\\ Y^{*}=Y_{\text{tot}}\frac{k_{\text{ry}}<R^{*}>+k_{y}}{k_{\text{ry}}R^{*}+k_{y}+k_{-y}}\\ X^{*}=X_{\text{tot}}\frac{k_{\text{sx}}S+k_{x}}{k_{\text{sx}}S+k_{x}+k_{\text{yx}}Y^{*}+k_{-x}} \end{array}$$

Assuming that all pathways are far from saturation, and all forward basal rate constants are zero, we can obtain transparent analytical results (see Appendix C). Firstly in the non-diffusible case, we find that

(21) $$\begin{array}{l} R^{*}=\frac{\alpha S}{1+\beta R_{0}} \end{array}$$

where *R*_0_ = [(1 + 4*αSβ*)^1/2^−1)/2*β*. Now, when the negative feedback element is diffusible, we get an identical expression, except for the fact that *R*_0_ = [(1 + 4*α*<*S*>*β*)^1/2^−1)/2*β*.

Both these expressions indicate that while negative feedback by itself tends to reduce the response locally and hence can be expected to reduce spatial variation in response to a spatial signal, a global negative feedback in fact accentuates the gradient response. This is also in contrast to the global positive feedback, which acted to smudge the response.

While we only concentrate on spatial signals in this paper it should be noted that a negative feedback network can also exhibit an adaptive (perhaps partial) response to homogenous signal inputs. Again this indicates how having a diffusible element leads to a qualitative difference in the spatial steady state response when compared to the temporal steady state response (the spatial response is accentuated and distinctly non-adaptive).

#### Biological relevance

The fact that a ubiquitously occurring feature such as negative feedback can have such a result suggests that cells might make use of negative feedback, or perhaps a multiplicity of negative feedbacks to achieve sharp spatial responses, and further that negative feedback may combine with the natural complexity of cell signalling networks to robustly organize spatial responses. Further such global negative feedback can combine adaptive (perhaps partial) response in temporal stimuli, with distinct non-adaptive response in spatial signalling.

Global negative feedback interactions are observed in calcium signaling pathways [37]. One example is when the G-Protein G_*q*_ activates PLC*β*which in turn hydrolyses PIP_2_ into Inositol-3,4,5-trisphosphate (InsP_3_). InsP_3_ stimulates release of Ca^2+ ^into the cytoplasm. Regulators of G Protein signaling (RGS) inhibit *G*_*q*_ signalling and are activated by high concentrations of Ca^2+ ^in the cytosol. A distinct example of the negative feedback network is in the EGFR autocrine signalling circuit, and has been used as a basis for modelling polarization responses [18].

### Cyclic module

We now examine the cyclic module: this module involves *n* species (labelled from 1 to n) in an irreversible cycle, with non-zero conversion rates. A signal, which is spatially inhomogeneous regulates the conversion of species 1 to 2. Every other conversion rate is spatially homogeneous. The case *n *= 2 reduces to a simple reversible reaction, with the signal regulating one reaction.

**Figure 6:**
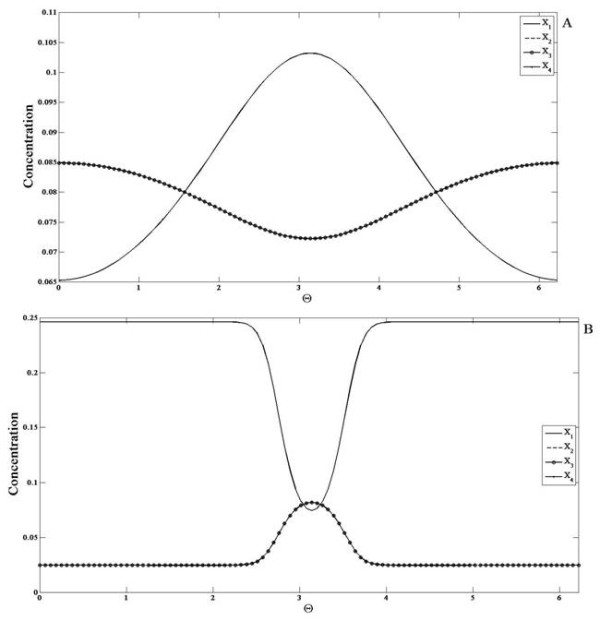
**Response of the cyclic reaction network module. ***X*_1_(solid line), *X*_2_(dashed line), *X*_3_(solid line with circles) and *X*_4_(solid line with dots) are shown for when the module (all elements non-diffusible) is subjected to a (**A**) gradient signal and a (**B**) localized signal input. Note that the profiles of *X*_2_, *X*_3_and *X*_4_are coincident for the parameters chosen. The concentration profile of *X*_1_is qualitatively opposite to that of the signal while *X*_2_, *X*_3_and *X*_4_are qualitatively similar to the input.

We first consider the response of the cyclic module to spatially varying signals with all species non-diffusible. For purposes of simulations, a cycle with *n *= 4 was considered. When a gradient signal is applied as mentioned, we find that at steady state, all the species except *X*_1_ show a graded response which is qualitatively the same (with same gradient features) as the external signal while *X*_1_ shows a graded response opposite to that of the signal. The same conclusions also hold good for a localized signal (with a background level which is non-zero) (see Figure 6).

**Figure 7:**
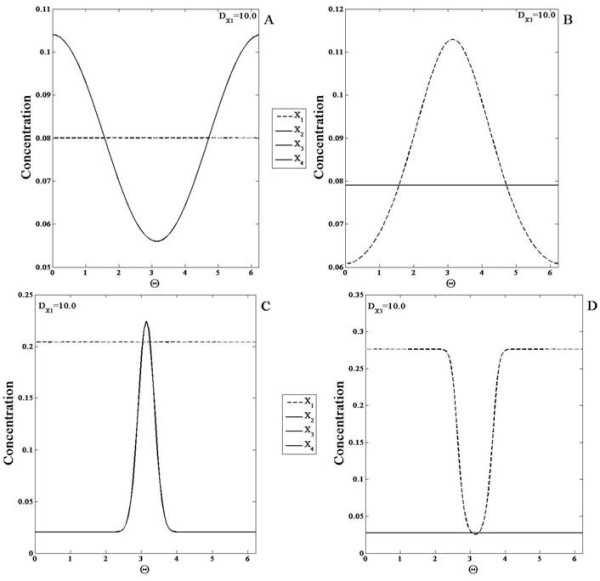
**The Cyclic Reaction network module with highly diffusible elements.** The response of the module to a (**A,B**) gradient signal and a (**C,D**) localized signal is shown when *X*_1_ is highly diffusible and when *X*_3_ is highly diffusible. Note that the profiles of *X*_2_, *X*_3_ and *X*_4_ are coincident for the parameters chosen. (**A,C**) If only *X*_1_ is highly diffusible, then *X*_1_(dashed line) is spatially homogenous and the remaining species (solid line) have a stronger spatial variation (than the corresponding non-diffusible case). (**B,D**) If only *X*_3_ is highly diffusible then *X*_2_, *X*_3_ and *X*_4_(solid line) are spatially homogenous and *X*_1_(dashed line) assumes a profile qualitatively opposite to that of the signal with stronger spatial variation than the non-diffusible case.

We now consider the possibility that one of the elements is diffusible. We consider this in two stages. In the first case *X*_1_ is highly diffusible. At steady state, *X*_1_attains a flat profile with no spatial variation, while the other elements all exhibit a graded response which mirrors that of the signal, but which is in some respects more pronounced than in the case of non-diffusible elements. Then we examine the case where one of the other elements is highly diffusible. From the structure of the network, we expect that if species *k* is diffusible then it will attain a flat steady-state profile, and all other species from *k* + 1 to *n* (i.e. downstream of it, and not directly regulated by the signal) will also attain homogeneous profiles at steady state. This is indeed what is observed. What is more interesting is the fact that all species from *2* to *k* also attain homogeneous profiles. In fact only species 1 exhibits an inhomogeneous profile (see Figure 7). In other words only the species directly regulated by the signal exhibits an inhomogeneous spatial profile at steady state, even if only one other species is highly diffusible. The analytical results for the cyclic network are shown in Appendix D, which also explain the reason for this result.

#### Biological relevance

We have analysed both a purely local cyclic network and a cyclic network with a highly diffusible/global component. An interesting insight revealed from our analysis was that when particular elements in the cyclic network are highly diffusible, both “upstream” and “downstream” elements show a spatially homogenous response to a spatial signal. This indicates that in such networks, if individual elements exhibit inhomogeneous spatial profiles then direct spatial regulation needs to be present at one or more locations in the cycle.

Cyclic networks form a part of the intracellular signalling network of the cell. Numerous examples of purely temporal cyclic networks such as the phosphotransferase system in *E. Coli* and metabolic cycles have been observed [45]. An example of such a network with a global element(s) is seen in the extracellular signal regulated endocytosis process [46]. In endocytosis, activated GPCRs on the plasma membrane are phosphorylated and form complexes. These GPCR complexes are transported (in vesicles pinched off from the plasma membrane) to compartments in the cytosol. From these compartments they are transferred to different compartments called recycling endosomes so that they can be transported back to the plasma membrane. Thus in this cycle, certain components of the network such as the GPCR are bound to the membrane and localized and other components such as the complexes being transported via endosomes form a part of a global pool in this network.

### Monostable switch module

Monostable switches may be realized in different ways, for instance through co-operative effects of regulating enzymes and combination of stage-wise effects such as in MAP Kinase cascades. Here we consider a commonly used model for monostable switches, namely the Goldbeter-Koshland model. The Goldbeter Koshland module contains two enzymes S and U which act close to saturation. In the module the signal regulates the enzyme catalyzing the forward reaction, and for simplicity, the signal is identified with this enzyme. We note that the steady state output of this module as a function of signal exhibits a switch-like response.

This can be understood intuitively in a straightforward manner. Both forward and backward reactions function essentially as zeroth order reactions, and are dependent on enzyme concentrations only. If the signal is weak enough so that the backward reaction is the stronger of the two, then the response is essentially zero at steady state. On the other hand when the signal exceeds a threshold, whereby the forward reaction is now stronger than the backward reaction, the output of the module is one corresponding to essentially complete conversion of reactant *X* into response *X*^∗^.

**Figure 8:**
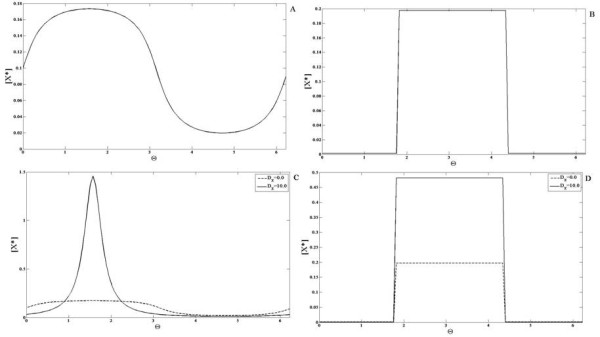
**Response of the monostable switch module. ***X*^∗^is shown in response to a smooth gradient signal (of the form *a* + *bsinθ*, with a maximum at *θ *=* Π*/2 ) for a (**A**) non-diffusible and (**C**) highly diffusible case and a localized signal input (of the form of a square pulse) for a (**B**) non-diffusible and (**D**) highly diffusible case. (**A**) The concentration profile of *X*^∗^is broader near the maxima and minima of the signal and it exhibits a spatial switch-like behaviour. (**B**) The spatial profile of *X*^∗^is qualitatively similar to the signal. (**C,D**) *X*^∗^is shown here for when *X* is highly diffusible (solid line) and non-diffusible (dashed line). (**C**) In response to the gradient signal, the spatial profile of *X*^∗^increases sharply in the region where the signal crosses a threshold value. Thus the diffusion of *X* leads to an enhancement of the spatial switch response. (**D**) For the localized signal, the spatial profile of *X*^∗^shows greater contrast when *X* is highly diffusible.

We first examine the case where none of the elements are diffusible. When a gradient signal input is applied to the module, the response exhibits this switch-like property in the spatial profile. This follows simply from the fact that in certain spatial regions, the signal is above the switch threshold and in others it is below the threshold. Thus such a binary computation is reflected in the spatial profile (Figure 8). An analogous result is seen in the case of a localized input for the same reasons.

Now we consider the case where one of the elements is highly diffusible. If *X*^∗^ is highly diffusible, then clearly at steady state it attains a uniform profile. It should be noted that this uniform level implicitly accounts for the region where the switch is on. We now consider the case where it is X which is highly diffusible. When a gradient signal is applied to the module, the steady state profile of *X*^∗^ appears to be very sharp in the region of the maximum (of the signal) and flattens out in the region of the minimum (Figure 8). In the case where a localized signal is applied to the module, the steady state concentration profile of *X*^∗^becomes sharply localized and it is sharper than the profile observed for *X*^∗^ when all species are non-diffusible. Thus the fact that *X* is diffusible in some respects enhances the effect of the switch.

These effects are examined in more detail in Appendix E. A comparison to other monostable switches is also outlined in Appendix E. The essential conclusion in all these cases is that by having the inactive form of the network element being highly diffusible accentuates the switching effect in spatial signalling, while having no effect in temporal steady state response.

#### Biological relevance

Having a global element relieves the constraint on the amplitude of the switch in spatial signalling. Monostable switches may be embedded in more complex networks where immobilization or sequestration of certain components works in conjunction with global elements to accentuate a switching response. A similar accentuation in signalling has been modellled in multiple contexts including Rho GTPase dynamics [17,47].

### Bistable module

#### Bistable module with weakly diffusing or non-diffusible entities

**Figure 9:**
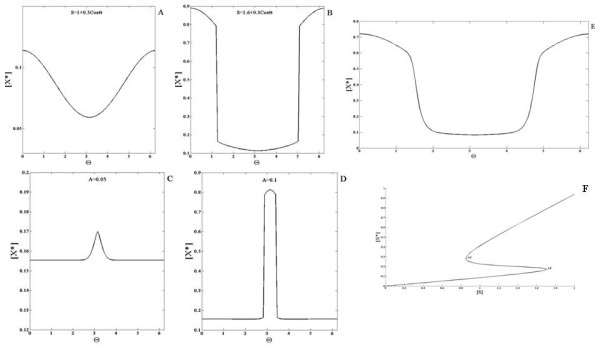
**Response of the mutual inhibition bistable switch module. ***X*^∗^(solid line) is shown when the module is subjected to (**A,B**) gradient signals and (**C,D**) localized signal inputs. (**A,C**) When the value of the signal in the entire domain is either above or below the critical value S2 (see text), the response is qualitatively similar to the signal. (**B,D**) A spatial switching effect is observed when the signal crosses the critical value in some part of the domain. (**E**) The above switching effects are seen even if some of the elements are weakly diffusible. Furthermore, even if the signal is entirely in the bistable regime, a spatial switching effect can be seen in the profile of *X*^∗^(with different initial conditions, see text). (**F**) A bifurcation diagram for this module is shown. The module has two stable steady states and one unstable steady state (between the two ’LP’ markers) in a signal range (S1 to S2, see text).

The bistable module has two elements X and Y that inhibit one another. This double negative feedback gives rise to the property of bistability. First we examine the case when none of the elements are diffusible. This module exhibits bistability for a range of input values ranging from *S*_1_ to *S*_2_[28]. Simulations are performed starting from a steady state corresponding to the “lower branch” in the bifurcation diagram (depicting X as a function of signal) for some basal value (Figure 9). Now if a gradient signal is applied where the local value of the signal never exceeds *S*_2_, then the steady state reflects a heterogeneity broadly mirroring the input (i.e. no switching effect). On the other hand if a gradient is chosen so that the local level exceeds *S*_2_, then a steady state is observed which reflects a spatial switch (Figure 9). Essentially, the part of the domain where the signal is below *S*_2_ends up at a steady state on the lower branch of the bifurcation diagram, while that part of the domain which is subject to a signal above the threshold evolves to the elevated, unique steady state. Thus the temporal steady state threshold behaviour is reflected directly in the spatial profile. It is also worth pointing out that this switching behaviour is observed whether the remaining part of the domain faces a signal completely in the bistable regime (i.e. *S*_1_ <* S *<* S*_2_) or even if some part of the domain is in the other monostable regime (i.e. *S *<* S*_1_).

It should be noted that here the dynamics and steady state is purely local and completely spatially decoupled (Incidentally it is worth pointing out that such a completely uncoupled bistable system possesses other steady states due to complete decoupling and locality, some of which are physically unreasonable). In such bistable systems, it is very important to check if the results in the strictly non-diffusible case carry through when species are weakly diffusible, and are not artifacts of a special situation.

Keeping this in mind, we performed simulations of the system, incorporating a weak diffusion of the element Y (similar results are obtained if X is weakly diffusing as well). What is observed may be summarized as follows. The results for the case where the input signal is such that part of the domain is above the threshold *S*_2_mirrors the non-diffusible case. A spatial switching behaviour is indeed seen here too. Again this is the case for gradient signals whether or not their lower limit falls below *S*_1_ (note that the initial conditions correspond to a steady state in the lower branch at a uniform signal value). This confirms the fact that this spatial switching behaviour is not an artifact of species being strictly non-diffusible.

Before discussing the diffusible case, we examine some additional cases. First we examine cases where the signal lies entirely in the bistable region (*S*_1_ <* S *<* S*_2_). While for the initial conditions used above, a gradient signal results in heterogeneity but no switching, this is no longer true if initial conditions are changed (different initial conditions reflect the prior signalling history in network components). Here if an initial condition is chosen such that part of the domain is at (or close to) the lower steady state branch, and the other part of the domain is at (or close to) the upper steady state branch, then it is possible for the system to exhibit spatial switching and a pulse like response (Figure 9). Thus the switching does not need part of the signal to be in a monostable regime. However, it should be noted that if the signal is spatially uniform, then the above simulation results in a spatially uniform steady state. This situation can be understood easily in terms of the Maxwell condition of uniform bistable systems [48]: generically one of the two steady states will “engulf” the other through travelling fronts which sweep across the domain. When the signal applied is a gradient, the situation is more subtle. If the heterogeneity is very weak, then the behaviour is similar to the homogeneous case, and a weakly heterogeneous steady state is obtained. On the other hand as the level of heterogeneity is systematically increased (and simulations performed with the same initial condition), one can observe the system evolve to a steady state which exhibits spatial switching. This effect is due to the fact that the fronts which tend to sweepacross the domain (as they would in homogeneous bistable media) get trapped in sites of heterogeneity–this is an effect called wave pinning and has been the focus of many studies (for eg. see [47]). Overall we see that the response of a bistable module with weakly diffusing elements to gradient signals is actually very subtle. In contrast to the temporal bistable case, we see a subtle interplay between the bistability, diffusion and signal heterogeneity as well as the previous history of the system.

#### Bistable module with strongly diffusing or global entities

**Figure 10:**
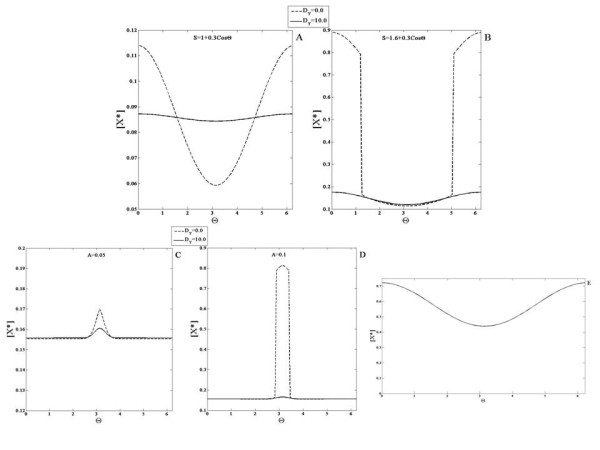
**The response of the mutual inhibition bistable switch module with highly diffusible elements.** The spatial profile of *X*^∗^is shown when *Y * is highly diffusible (solid line) and non-diffusible (dashed line) for (**A,B**) gradient signals and (**C,D**) localized signal inputs. When *Y * is highly diffusible, the switch-like behaviour observed in the non-diffusible case is no longer present. (**E**) This plot illustrates that the system can achieve a higher steady state value (with suitable initial conditions) without exhibiting spatial switching behaviour when the signal is in the bistable regime.

We now turn to the case where one of the elements (Y) is highly diffusible (Figure 10). We note at the outset that the high diffusivity of Y does not affect the bistability feature of the module. Indeed in spatially homogeneous signals, the module can exhibit multiple steady states. When the module is subject to a gradient, what is observed is as follows. For a gradient signal where part of the signal crosses the threshold *S*_2_, we find that in contrast to the earlier case, no spatial switching is observed. In fact a heterogeneous profile roughly reflecting the heterogeneity of the input is seen. The bistable character of the network is reflected in the fact that (depending on initial conditions), the system can evolve to the heterogeneous “equivalent” of the multiple stable temporal steady states. These results are explained analytically in Appendix F.

In summary, in the highly diffusible case, the response of the module results in multiple heterogeneous steady states but without any spatial switching.

In this subsection we have examined a type of positive feedback network which arises from a mutual inhibition network which exhibits the property of bistability. A spatial switch response is exhibited by a purely temporal network– this response appears in certain domains. Furthermore the spatial switching response has a dependence on the prior state of the system or its history. When one of the interacting elements is highly diffusible the spatial switch is no longer realized by the network. In other words having a global element in the feedback acts as a constraint as far as realizing spatial switching. Thus whether or not a spatial switch could be realized through the bistability feature is more complex than a monostable or simple temporal bistable switch.

#### Biological relevance

These results have interesting implications for the organization of networks where spatial switches are observed. Firstly bistable circuits may be able to act as spatial switches if all elements are weakly diffusible (or non-diffusible). If a global element is present in the feedback circuit giving rise to bistability, then this circuit by itself cannot give rise to spatial switching. In this case it may be that some upstream signalling actually provides the spatial switching, which is partly accentuated by the bistable circuit. The other possibility is that some sequestration mechanism or additional pathway (non-global) is responsible for realizing the spatial switch through bistability. Finally, our analysis above is based on elements where the active and inactive forms are both highly diffusible. If only the inactive form of such an element is highly diffusible, then this can play a role in accentuating the switching effect similar to the analysis in the monostable switch module section.

While bistability has been observed and studied in detail in temporal signalling, it has also been postulated to be the basis of spatial organization of Rho-GTPases in polarization [47] and to give rise to phosphoprotein waves in protein kinase cascades [49].

### Negative feedback oscillator module

We now examine a module where the asymptotic state may not be steady, and instead result in sustained oscillations. This is an oscillator module, and for specificity, we consider a negative feedback oscillator module. The negative feedback oscillator module consists of three species *X, Y * and *R*, where Y is the intermediate negative feedback element and *R*^∗^ is the response. In this module, there is a critical value of the signal where a supercritical Hopf bifurcation occurs, leading to sustained oscillations.

**Figure 11:**
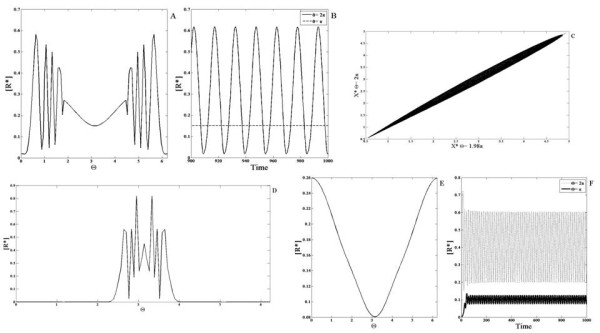
**Response of the negative feedback oscillator module.** The response *R*^∗^(solid line) is shown for a (**A, B, E** and **F**) gradient signal and a (**D**) localized signal input. (**A,D**) The profiles are shown at a snapshot in time. *R*^∗^exhibits irregular spatial oscillations when the signal is in the oscillatory regime and the profile is smooth in regions where the signal is outside of this critical range. (**B**) This plot illustrates the variation of concentration of the response with respect to time at two spatial locations: the middle of the domain (dashed line) and at the boundary (solid line). The response only exhibits temporal oscillations in the regions where the signal is in the critical oscillatory range. (**C**) The phase plot is shown for *X*^∗^at two adjacent points in the domain. It depicts a densely filled region highlighting the quasi-periodic nature of oscillations. (**E**) When *Y * is weakly diffusible, the spatial profile of the response becomes smooth and (**F**) periodic oscillations in time are observed in the middle of the domain (solid line) whereas in the non-diffusible case they are absent in that region (as seen in (B)).

We first assess the case when none of the elements are capable of diffusion. When a gradient signal is applied- with some region of the signal above the critical value for oscillations and some below it, the spatial concentration profile of *R*^∗^shows irregular spatial oscillations for regions in space where the signal is in the region past the Hopf bifurcation and a smooth static profile when the signal is below it. The spatial concentration profiles of *R*^∗^ are shown for a snapshot in time in Figure 11.

When the gradient signal is completely above the bifurcation threshold (but within the oscillatory regime), then the entire profile of *R*^∗^oscillates irregularly with respect to space. For both these gradient inputs, the concentration of *R*^∗^ shows irregular spatial oscillations (in the appropriate regions) as well as regular oscillations in time for these spatial regions. To obtain a clearer picture of the behaviour of the module, we created a phase plot of the concentration of the response at adjacent spatial locations. The phase plot shows a region which is essentially densely filled. This indicates the source of the irregular spatial oscillations: at any spatial location, the behaviour is one of regular temporal oscillations (in the regions which lie in the oscillatory regime). However, owing to the spatially varying signal, and complete spatial decoupling, different regions experiencing different signal values exhibit oscillations whose frequencies are not rationally commensurate with one another and this results in quasiperiodic behaviour of the distributed dynamical system. Similar insights hold good for localized inputs. This indicates that a simple spatial biasing of an oscillator circuit does not lead to regular spatial oscillations.

Now in the above case, if the elements are made diffusible, but relatively weakly so, this can cause an effective coupling between different regions. The case where some part of the domain is in the oscillatory regime is shown in Figure 11. We now find a smoother spatial profile is observed, in this part of the domain. Typically as the diffusion is made stronger (but still relatively weak) , we find stronger spatial coupling and spatial (and temporal) oscillations, which lie outside the part of the domain in the oscillatory regime.

**Figure 12:**
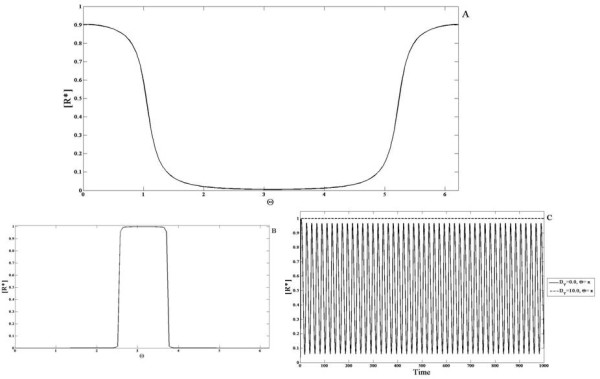
**The effect of diffusible species in the response of the negative feedback oscillator.** The response is shown when the feedback element is highly diffusible (solid line) for a (**A**) gradient signal and (**B,C**) localized signal input. (**A,B**) A smooth concentration profile qualitatively similar to the signal is observed. This is because the diffusion of the feedback element acts to couple the different regions of the domain resulting in such a profile. (**C**) The temporal profile of the response is shown when *Y * is highly diffusible (dashed line) and non-diffusible (solid line), corresponding to the signal in (**B**). For the non-diffusible case oscillations are observed in the middle of the domain. However when *Y * is highly diffusible periodic oscillations are not observed in this region because the spatial average of the signal is not in the oscillatory regime. Thus, an heterogeneous, static profile emerges in the diffusible case.

Finally we analyze the case when *Y * is highly diffusible (Figure 12). When a gradient signal is applied to the module and even if it is above the Hopf bifurcation value (i.e. oscillation threshold) the profile of *R*^∗^is smooth. A phase plot of the concentration of the response at two adjacent spatial points no longer shows a filled region but a closed curve indicating that the oscillations at different points in space are now synchronized with one another. Similarly when a localized signal is applied, the profile of *R*^∗^ becomes smooth and amplitude of the response increases. It is worth pointing out that when the element Y is highly diffusible, this plays a role in effectively coupling different regions. Thus in this situation, we find that if the spatial average of the signal is essentially in the oscillatory regime then the entire domain oscillates in time, with spatial heterogeneity being reflected in the non-diffusible elements. On the other if the spatial average of the signal is below the threshold for oscillation, the system attains a heterogeneous steady state, even though there are regions which are locally in the oscillatory regime.

In summary, spatial biasing of an oscillator circuit can lead to oscillatory responses in different spatial regions which are regular, provided some elements are diffusible (not necessarily strongly). Having a diffusible element strongly couples different elements and results in either the entire domain oscillating or the entire domain being static.

#### Biological relevance

The above results have implications for the organization of spatial oscillators (inhomogeneous regions in space which exhibit temporal oscillations) in cells. While spatial oscillators can be generated by biasing of an oscillator circuit with (not too strongly) diffusible elements, having highly diffusible elements in the circuit generating oscillations result in complete global coupling. It follows that a way in which cellular signalling networks may be organized for this purpose is to have parallel pathways/modules to provide spatial variation/localization, and an oscillator module with diffusible/global elements to provide global oscillation, which are then combined. Such a combination could give rise to co-ordinated/synchronized oscillations in different regions of the cell (for instance two poles of a migrating cell). Another way to have co-ordinated oscillations in different spatial regions is by having a circuit which generates oscillations in a relatively localized region, which is then communicated to other regions by diffusible/global intermediates. It is worth pointing out that fully self-organized spatial oscillations may also occur, and it remains to be seen how spatial oscillations in different cell types may be organized and generated.

Finally the analysis of another kind of switch module which involves positive feedback and exhibits a transcritical bifurcation is discussed in Appendix G. The essential conclusion from this analysis is also that if the feedback element is highly diffusible, then the particular switching behaviour in spatial gradients may be abolished, even though this switching continues to be observed in temporal signalling.

## Conclusions

The past decade has seen a significant amount of work in understanding temporal aspects of cellular signal processing, both in specific contexts, and also conceptually. However in many of these studies spatial aspects of signal processing are often ignored, typically on the grounds that any key aspects of signalling can be captured in appropriate temporal models. However, even here, it is increasingly being recognized that the spatial dimension to signalling contains highly non-trivial, and in some cases vital information which provides key insights into the signal processing (for example, it has been suggested that spatial gradient information of particular entities in fission yeast is a key link which couples growth and the cell cycle [50]). In cellular processes such as cytokinesis, eukaryotic chemotaxis, wave-propagation in the cell, spatial aspects of signalling cannot be bypassed. The spatial aspect presents extra challenges for both modelling as well as experimental elucidation of the system. It is being increasingly recognized that spatial aspects of signalling need to be seriously addressed, and that in this regard frameworks which provide conceptual understanding of relevant issues are necessary.

At the outset, we note that spatially non-trivial behaviour in signalling networks could arise, either because some external or “upstream” signals in a network are heterogeneous, or because some of the elements in the network are themselves heterogeneously distributed, or if there is some spontaneous pattern-forming process. We focus on the first case here, and will address other issues separately. We note that spatial variation in a number of signalling entities may be generically expected in cellular environments.

Our focus was on spatial aspects of signal processing, in particular with a view towards determining whether qualitatively new features emerge when one includes spatial considerations. This dictated our approach in this paper: rather than focus on mechanistic modelling, we focussed on representative network modules of signalling and considered these in a spatially extended domain, with spatially inhomogeneous signals, both gradual and sharp, of the kind which may be generically expected. While our modelling was performed in 1-D in a periodic domain for specificity, most of the results carry through for other boundary conditions, and more complicated domains. Since signal processing in these networks could be significantly affected by diffusion, we considered the effect of diffusional transport in these modules, by making different elements highly diffusible. Having elements being highly diffusible can fundamentally affect the signal transduction in a network, and indeed the classic work of Turing (and others) shows how diffusible elements can even drive spatial pattern formation, in essentially homogeneous backgrounds. By studying spatial signalling in representative modules with and without diffusion, we provide a basis for understanding how different types of typical signal transduction behaviour is realized spatially. We also believe that by considering a variety of modules together, we are able to compare these effects between different modules.

While we adopt a more general approach, we mention that many of the effects and networks we study are seen in different signalling contexts. Spatial variation of signalling entities/inputs can be generically expected in many situations, and the signalling network behaviour we investigate is repeatedly observed. Key second messengers like cAMP and cGMP are highly diffusible, and these (along with appropriate compartmentalization) regulate a number of effectors and this allows for chemical circuits to naturally include global effects [1]. This is also true when the driving signal (say on the cell membrane), leads to signal transduction, involving cytosolic elements, which are not directly (locally) regulated by spatially inhomogeneous signals. The presence of cytosolic pools can provide the kind of the global effect studied here. Signal transduction through phosphoinositides and other species on the membrane, leads to the recruitment of cytosolic elements such as PKA (regulated by cAMP) and PKC [51], which interacts with these networks, providing examples of global regulators in signalling networks. Other examples include the role of *IP*_3_ and calcium signalling providing global regulation and feedback effects [37]. The role of spatial effects and diffusion has been receiving attention and is relevant to G-protein cycles, spatial switches, polarity circuits, spatially organized oscillating elements (eg. regulation of actin in chemotaxing cells), and wave propagation [6-8,17,52-54]. Highly diffusible entities and global effects have thus been incorporated in a wide range of modelling efforts (for eg. see [27,55]). Thus we expect that the analysis performed here can provide valuable insights into the potential complexities of spatial signalling in multiple contexts and the role of global regulators therein. We consolidate our findings and end with a discussion on the insights gained from our results and implications for intracellular spatial signal processing.

### Spatial signalling with non-diffusible elements

**Figure 13:**
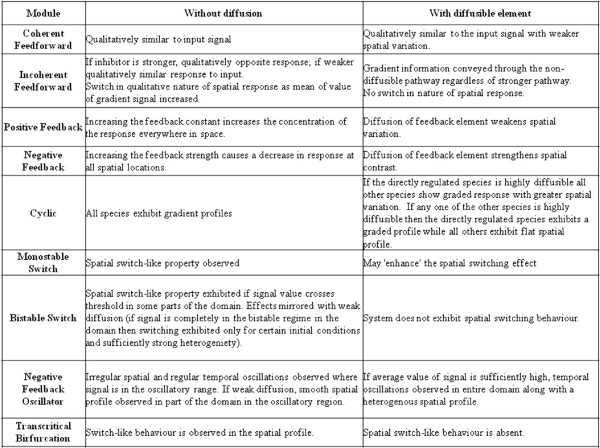
**Results from the analysis of the models.** A summary of results is presented in the above table, for steady state signal transduction (except for oscillator module).

The work presented here focussed on the behaviour of the modules (Figure 13) which have a natural input and output. When we examine the response of the modules (with all elements non-diffusible) to spatial gradient inputs, the output is typically a spatial signal which reflects the steady state temporal signal processing. Indeed the signal processing here is purely local, spatially, with no coupling. This is examined firstly because it is the most basic way in which spatial signal processing can occur, and also because it is the basis for understanding spatiotemporal signal transduction (addressed subsequently). The coherent/incoherent feedforward modules, simple negative and positive feedback modules and cyclic modules all reflect their temporal signalling features in steady state spatial signalling. Likewise different monostable switches as well as switches with a transcritical bifurcation structure, reveal their switch like behaviour in spatial signal processing. This behaviour continues to hold good when any of the elements are weakly diffusible.

In the case of bistable switches if the signal range straddles the location(s) where switching occurs, then it is possible to observe spatial switching behaviour, and this continues to hold good even if different elements are weakly diffusible. On the other hand if the signal range lies entirely within the bistable regime, then it may or may not be possible to observe a spatial switching behaviour at steady state. From the perspective of spatial signalling this involves a subtle interplay between the bistability and the heterogeneity (such switching would not be observed in the absence of heterogeneity, even with bistability, if any of the elements were diffusible), and further depends in a subtle way on the history of how the gradient signal was imposed. This underscores various subtleties in understanding spatial switching in such a module, when compared to temporal signalling. In the case of an oscillator module, when subject to a gradient, we find that if the elements are non-diffusible, then temporal oscillations are observed in appropriate spatial regions, which are however spatially irregular. This is because different locations respond to local signal values which are different and not generally rationally commensurate. This leads to quasiperiodic behaviour in the spatiotemporal response. However in the case of quite weak diffusion, the coupling due to diffusion leads to a regular spatiotemporal oscillatory behaviour in appropriate regions.

### Effect of highly diffusible elements

In general, we find significant differences when highly diffusible elements are present in various modules. Thus, in the case of the incoherent feedforward module, we see that whatever the relative balance of the two competing pathways, the net spatial response is determined by the non-diffusible pathway, even if it is the weaker pathway kinetically. Thus the feature of having one pathway diffusible, allows for exact, or imperfect adaptation in homogeneous stimuli, along with a robust spatial response. Similarly, analysis of a different incoherent feedforward module indicates how having one pathway diffusible prevents a reversal in gradient response, as the mean value of the signal is changed. In the positive feedback module, if the feedback element was diffusible, then this resulted in the elevation of the response everywhere in the domain, including locations which were not subject to signals. In the negative feedback module, the high diffusivity of the feedback element results in a greater spatial contrast of the response. This, along with the fact that the feedback module can result in partial adaptation to homogeneous stimuli means that this module could be viewed as a feedback equivalent of a feedforward Local Excitation Global Inhibition module [38]. In an irreversible cyclic module, having a highly diffusible element results in spatially homogeneous profiles for all elements except that being directly regulated by the gradient signal: thus interestingly even elements “upstream” of the diffusible element attain spatially homogeneous profiles. The case of a reversible reaction with one element diffusible is a special case of this cyclic module.

Examining simple switches we find that if the inactive form of the response element is highly diffusible, then this accentuates the switching effect and leads to a larger amplitude switch, when compared to the non-diffusible case. In the case of a monostable switch realized through positive feedback (without cooperativity) resulting in a transcritical bifurcation, we find that having feedback mediated by a diffusible species abolishes the possibility of a spatial switch. It should be noted that the essential bifurcation behaviour of the signalling network continues to be reflected in its response to homogeneous signals of different strengths. Thus the switching effect of the signalling is present in a gradient, not through the manifestation of a switch in the spatial profile, but rather through a switch in the nature of the response (whether completely switched off or not) which in turn depends on the average of the signal. A very similar effect is seen in bistable switches. When the feedback element is highly diffusible, a steady state response possessing a switching effect in the spatial profile is not seen. Instead the bistability is reflected in the fact that (depending on signal ranges) it is possible to have multiple stable spatially graded responses, which may be seen as spatial analogues of the temporal behaviour of the module. The effect of highly diffusible elements in oscillatory modules serves to effectively couple different regions of the domain to produce spatiotemporally oscillatory behaviour. Thus strong diffusion allows regions which are faced with signals not strong enough to excite oscillations, to oscillate robustly, and in other cases have the opposite effect.

### Spatial and temporal signal processing

Our analysis of these modules provides insights into the signal processing capabilities of these modules and the constraints with regard to spatial signalling imposed by diffusion. Taken together, we can say that having highly diffusible or global elements, either completely blocks propagation of spatially varying signals and communicates only the average effects of the upstream signal, or leads to significant distortions in signal propagation. Typically having key elements being highly diffusible causes fundamental differences in steady state spatial responses: switch effects are not realized, adaptation does not occur in spatial signals, oscillations are promoted or obstructed in particular regions, redundancies in signalling are removed. Whether this is a fundamental advantage or a constraint depends on the context. The temporal dynamics and bifurcation structure of the model continues to be present and is reflected in the module response to homogeneous signals, and also in gradients (especially weak gradients). In other cases, high diffusivity can promote effects in signal processing like an increased amplification of switching, by essentially making available a larger pool for an elevated response. This also suggests how cells may employ appropriate mechanisms of localizing proteins (for eg. through scaffolds) to effectively combine with diffusible entities to result in spatial switching effects. More generally our analysis provides insights into the extent to which particular temporal signalling characteristics in networks could be translated into spatial signalling characteristics, the constraints involved, and suggests some ways in which the networks may be organized to realize particular spatial signal transduction patterns by conforming to these constraints or bypassing them using the natural machinery and elements of signal transduction available.

Our studies have focussed on a series of modules which act as representative elements for particular behaviour. For the most part, for the kind of analysis performed here, the main insights also carry through to other representative models. Thus a model of mutual activation with positive feedback [28], leading to bistability, exhibits similar behaviour to the mutual inhibition module for the kind of analysis performed here. A similar conclusion can be drawn for different classes of monostable modules. In this paper the spatial signals considered have been relatively simple but typical. We have not considered any spatiotemporal signal inputs, and will examine these issues in detail subsequently. We will also examine more complex network structures in detail later. Likewise, for all the network modules we have considered, inspite of the strong non-linearity the resulting spatial pattern was driven by the input signal to the module. Thus, we have not dealt with genuine symmetry breaking and pattern-formation in the spatial signal processing. This again will be dealt with subsequently.

Overall we have taken the first step towards a network-based exploration of spatial signalling (whose insights extend beyond intracellular signalling), and a systematic study of the role of global elements therein. The analysis indicates various surprising effects which arise in spatial signalling arising from a complex combination of non-linearity, diffusion and heterogeneity and how they may be underlying signalling patterns in basic modules. These results provide a basis for both understanding particular patterns of chemical signal propagation where spatial effects are important, and also a way for starting to build synthetic circuits in the cell which harness spatial signal transduction in a substantial and non-trivial way [56-58].

### Appendix A: Perturbation analysis

The derivation of the perturbation analysis which is used in the coherent feedforward model is discussed in this section. We obtain a basic result which is used elsewhere in the text.

In the text, we were concerned with the response of basic reversible reaction system (species X) to a signal S, when the active and inactive forms were highly diffusible.

The steady state of the element *X*^∗^is obtained from equation (12), and satisfies the equation

(22) $$\begin{array}{l} (k_{\text{sx}}S(\theta )+k_{x})(X_{\text{tot}}-X^{*})-k_{-x}X^{*}+D_{X}\frac{\partial^{2}X^{*}}{\partial \theta^{2}}=0 \end{array}$$

We will determine the steady state of *X*^∗^ from above, in the limit of high diffusion coefficient *D*_*X *_using a perturbation expansion, and make use of this result in all the modules.

Expanding the variable *X*^∗^in powers of *ε *= 1/*D*_*X *_as

(23) $$\begin{array}{l} X^{*}=X_{0}+\varepsilon X_{1}+\varepsilon^{2}X_{2}+\mathrm{..} \end{array}$$

and substituting the above equation and collecting like powers of *ε* we have

(24) $$\begin{array}{r} \frac{\partial^{2}X_{0}}{\partial \theta^{2}}=0\\ \frac{\partial^{2}X_{1}}{\partial \theta^{2}}+(k_{\text{sx}}S(\theta )+k_{x})(X_{\text{tot}}-X_{0})-k_{-x}X_{0}=0 \end{array}$$

From the first equation, applying periodic boundary conditions, we immediately see that *X*_0_ is a constant. The value of the constant is determined as a solvability condition from the second equation by integrating the second equation from 0 to 2*Π* and dividing the resulting equation by 2*Π*. It immediately follows that

(25) $$\begin{array}{l} X_{0}=X_{\text{tot}}\frac{k_{\text{sx}}<S>+k_{x}}{k_{\text{sx}}<S>+k_{x}+k_{-x}} \end{array}$$

where <*S*> denotes the spatial average of S over the domain defined as

(26) $$\begin{array}{l} <S>=\frac{1}{2\Pi }\int_{0}^{2\Pi }S(\theta )d\theta \end{array}$$

The above expression simply reveals that for highly diffusible components, the steady state is essentially uniform at a level dictated by the spatial average of the signal.

This basic result is employed in other modules as well.

### Appendix B: Analytical results in the positive feedback model

The steady states for the positive feedback model were considered in the text. The solutions of the above equation are more transparent for certain special cases, which are discussed here. Thus we consider the case *k*_*r *_=* k*_*y *_=* k*_*x *_= 0. Furthermore, we can simplify matters by assuming that each of the enzymes at steady state has a concentration which is not close to the maximum available enzyme. From this second assumption, we can neglect the terms in the denominator which correspond to the numerator. With these further simplifications, we can obtain the equations, for the case of non-diffusible elements

(27) $$\begin{array}{c} \;R^{*}=R_{\text{tot}}\frac{k_{\text{xr}}X^{*}}{k_{-r}}\\ Y^{*}=Y_{\text{tot}}\frac{k_{\text{ry}}R^{*}}{k_{-y}}\\ \;\;X^{*}=X_{\text{tot}}\frac{k_{\text{sx}}S+k_{\text{yx}}Y^{*}}{k_{-x}} \end{array}$$

This can be solved to obtain the response from the equation *R*^∗^ =* αS* + *βR*^∗^, where *α *=* k*_*xr*_*k*_*sx*_*X*_*tot*_*R*_*tot*_/(*k*_−*r*_*k*_−*x*_) and *β *=* X*_*tot*_*Y*_*tot*_*X*_*tot*_*k*_*xr*_*k*_*yx*_*k*_*ry*_/(*k*_−*r*_*k*_−*x*_*k*_−*y*_). Note that the feedback constant is contained in the parameter *β*. Now from this equation, the solution for the response is given by

(28) $$\begin{array}{c} R^{*}=\frac{\alpha S}{1-\beta } \end{array}$$

This clearly shows that as the feedback constant increases, so does the response. Note that as *β* starts to get comparable to 1, this above solution becomes a less accurate approximation of the real solution, and then saturation effects need to be taken into account.

We can now use this simplification to examine the case of a highly diffusible Y. In this case, the only equation which changes is that of *Y * which is proportional not to the local *R*^∗^ but to the spatial average of *R*^∗^. This leads to the equations

(29) $$\begin{array}{c} R^{*}=R_{\text{tot}}\frac{k_{\text{xr}}X^{*}}{k_{-r}}\\ \;Y^{*}=Y_{\text{tot}}\frac{k_{\text{ry}}<R^{*}>}{k_{-y}}\\ \;X^{*}=X_{\text{tot}}\frac{k_{\text{sx}}S+k_{\text{yx}}Y^{*}}{k_{-x}} \end{array}$$

This results in an equation of the form *R*^∗^ =* αS* + *β*<*R*^∗^> with *α *and *β *just as above. This is solved by averaging both sides of the equation with respect to space, to give <*R*^∗^> =* α*<*S*>/(1−*β*) and is of course valid only when *β* is clearly less than 1. As *β *starts to become comparable to 1, we immediately find that the assumption that most of the enzymes are present in inactive form starts to break down. From this, we find the response to be

(30) $$\begin{array}{c} R^{*}=\alpha S+\frac{\beta }{\left(1-\beta \right)}\alpha <S> \end{array}$$

We see that the feedback (contained in the parameter *β*) has a role in increasing the second term and hence increasing the level everywhere.

### Appendix C: Analytical results in the negative feedback model

The steady states of the negative feedback model are presented in the text. We present some results which extend the analysis and make it more transparent. While one can infer the effect of negative feedback from the results in the text, the effects become transparent, if we assume that every reaction is far from saturation at steady state. In this case, for non-diffusible Y, the steady state is given by

(31) $$\begin{array}{c} \;R^{*}=R_{\text{tot}}\frac{k_{\text{xr}}X^{*}}{k_{-r}}\\ Y^{*}=Y_{\text{tot}}\frac{k_{\text{ry}}R^{*}}{k_{-y}}\\ \;X^{*}=X_{\text{tot}}\frac{k_{\text{sx}}S}{k_{\text{yx}}Y^{*}+k_{-x}} \end{array}$$

From this the response is obtained as the solution of the equation

(32) $$\begin{array}{c} R^{*}=\frac{k_{\text{sx}}X_{\text{tot}}R_{\text{tot}}k_{\text{xr}}S/k_{-r}}{k_{-x}+k_{\text{yx}}Y_{\text{tot}}k_{\text{ry}}R^{*}/k_{-y}} \end{array}$$

It is easy to see that this equation is of the form

(33) $$\begin{array}{c} R^{*}=\frac{\alpha S}{1+\beta R^{*}}\\ \;\;\;\alpha =k_{\text{sx}}X_{\text{tot}}R_{\text{tot}}k_{\text{xr}}/k_{-r}k_{-x}\\ \;\;\;\beta =k_{\text{yx}}Y_{\text{tot}}k_{\text{ry}}/(k_{-y}k_{-x}) \end{array}$$

It is easy to see that the equation, when rearranged, leads to a quadratic equation, which has only one positive root. The effect of the negative feedback strength is also immediately seen. The feedback constant is contained in the parameter *β* and it is a simple matter to see that as *β *increases, the steady state decreases (the steady state being the intersection of two functions of *R*^∗^representing the LHS and the RHS, with the RHS representing a curve which decays faster as *β *increases).

This above approach can now be used to examine the case where Y is highly diffusible. In this case, with the above approximations, we see that

(34) $$\begin{array}{c} R^{*}=R_{\text{tot}}\frac{k_{\text{xr}}X^{*}}{k_{-r}}\\ \;Y^{*}=Y_{\text{tot}}\frac{k_{\text{ry}}<R^{*}>}{k_{-y}}\\ \;X^{*}=X_{\text{tot}}\frac{k_{\text{sx}}S}{k_{\text{yx}}Y^{*}+k_{-x}} \end{array}$$

Thus the steady state emerges as the solution of the equation

(35) $$\begin{array}{c} R^{*}=\frac{\alpha S}{1+\beta <R^{*}>} \end{array}$$

with *α *and *β *defined exactly as above.

Even without solving this equation, we can transparently see the effect of the global negative feedback in the denominator. The solution is completed by averaging both sides, and recognizing that the average of the response satisfies the equation

(36) $$\begin{array}{c} <R^{*}>=\frac{\alpha <S>}{1+\beta <R^{*}>} \end{array}$$

Again, the average of *R*^∗^satisfies the same equation (with respect to the average of S) as *R*^∗^ (with respect to S) in the non-diffusible case. Calling this solution *R*_0_ = [(1 + 4*α*<*S*>*β*)^1/2^−1)/2*β*, the solution for the response is given by

(37) $$\begin{array}{c} R^{*}=\frac{\alpha S}{1+\beta R_{0}} \end{array}$$

We see that the net dependence of the response on the signal is through a direct local regulation, which is damped through a global negative feedback.

### Appendix D: Analytical results of the cyclic model

We discussed the results of the cyclic model, presenting results for the cases where all elements were essentially non-diffusible and also for the case where a highly diffusible element may be present. We complement those results with detailed analytical results which explain the trends observed.

The numerical results obtained in the cyclic network can be understood analytically as follows. Firstly in the non-diffusible case we have

(38) $$\begin{array}{l} k_{1}S(\theta )X_{1}=k_{2}X_{2}\\ \;\;k_{i}X_{i}=k_{i+1}X_{i+1}(i=2,3,\mathrm{..}n-1)\\ \;k_{n}X_{n}=k_{1}S(\theta )X_{1} \end{array}$$

This is supplemented by a conservation condition *X*_1_ + *X*_2_ + *..X*_*n *_=* X*_*tot*_. This results in the solution

(39) $$\begin{array}{l} \;F=\frac{X_{\text{tot}}}{1/(k_{1}S(\theta ))+\overset{i=n}{\underset{i=2}{\sum }}(1/k_{i})}\\ \;X_{1}=F/k_{1}S(\theta )\\ \;X_{i}=F/k_{i}(i\geq 2) \end{array}$$

This equation readily reveals the nature of spatial variation of the various entities. Now we consider the effect of diffusible entities. We first consider the case that species 1 is diffusible. Then it follows from arguments similar to previous sections that

(40) $$\begin{array}{l} k_{1}<S>X_{1}=k_{n}<X_{n}>\\ \;k_{1}X_{1}S(\theta )=k_{2}X_{2}\\ \;\;k_{i}X_{i}=k_{i+1}X_{i+1}(i=2,3,\mathrm{..}n-1) \end{array}$$

This is supplemented by the equation <*X*_1_ + *X*_2_ + *..X*_*N*_> =* X*_*tot*_. This arises from the conservation of total amount of all species (note that since some species are diffusible, the local total amount of species is not conserved, but the amount averaged/integrated over the domain is conserved). The solution of the above equations is given by

(41) $$\begin{array}{l} X_{1}=\frac{X_{\text{tot}}}{1+k_{1}<S>\overset{i=n}{\underset{i=2}{\sum }}\frac{1}{k_{i}}}\\ \;X_{i}=k_{1}X_{1}S(\theta )/k_{i}(i=2,3,\mathrm{..}n) \end{array}$$

The clear spatial biasing of the species *i* is seen here.

We now turn to the case where species k is diffusible (*k *> 1). Clearly at steady state the concentration profile of *X*_*k*_ will be spatially uniform, and since *k*_*i*_*X*_*i *_=* k*_*i* + 1_*X*_*i* + 1_, for *i *>* k* all species *i *>* k *will have a spatially uniform profile as well. However if we consider species *i *= 2,3*.k*−1, from a mass/flux balance for the combination of species *j*,*j* + 1*..n*,1,*..i*, where *j *>* k *(all these species being non-diffusible, the net flux in has to equal the net flux out) we find that *k*_*i*_*X*_*i *_=* k*_*j*_*X*_*j*_ and since *X*_*j*_ is uniform, so too is *X*_*i*_. In this case we have the situation where species 1 is the only species with a spatially varying profile. Solving the equations, and using the conservation condition just as above yields

(42) $$\begin{array}{l} \;F=\frac{X_{\text{tot}}}{\frac{1}{k_{1}<S>}+\overset{i=n}{\underset{i=2}{\sum }}\frac{1}{k_{i}}}\\ \;\;X_{1}=F/k_{1}S(\theta )\\ \;\;X_{i}=F/k_{i} \end{array}$$

We finally conclude by noting the special case *n *= 2 which is that of a reversible reaction. We observe from above that if *X*_1_ is diffusible, then *X*_2_ has the spatial variation, which now has a somewhat different dependence on the signal when compared to the non-diffusible case. This is seen from the expression

(43) $$\begin{array}{l} X_{1}=\frac{X_{\text{tot}}}{1+\frac{k_{1}<S>}{k_{2}}}\\ X_{2}=\frac{k_{1}S(\theta )}{k_{2}}\frac{X_{\text{tot}}}{1+\frac{k_{1}<S>}{k_{2}}} \end{array}$$

Likewise, when species 2 is diffusible it will not have any spatial variation, and species 1 has a spatial variation inverse to the signal. This results in

(44) $$\begin{array}{l} X_{1}=\frac{1}{k_{1}S(\theta )}\frac{X_{\text{tot}}}{\frac{1}{k_{1}<S>}+\frac{1}{k_{2}}}\\ X_{2}=\frac{1}{k_{2}}\frac{X_{\text{tot}}}{\frac{1}{k_{1}<S>}+\frac{1}{k_{2}}} \end{array}$$

Both these expressions transparently demonstrate how the high diffusivity of one element can affect the response. In summary we see how the high diffusivity of particular elements in the cycle, has the effect of spatially homogenizing both downstream and upstream elements in the cycle.

### Appendix E: Analytical results of the monostable switch model

In this section we will examine the results from the monostable switch model in a little more detail. A comparison to other monostable switch models is also drawn here.

We consider the case where the signal is such that the forward reaction is stronger than the backward reaction when *θ*∈[*θ*_1_,*θ*_2_]. The case where all elements are non-diffusible would reveal that essentially, the response *X*^∗^ =* X*_*tot*_ in this region and is 0 elsewhere. Note that *X*_*tot*_sets a maximum locally attainable value for the response.

When *X*^∗^ is the diffusible element then it is easy to see that it will attain a spatially homogeneous profile (and can be understood similar to the analysis below). The more interesting case to consider is the situation where *X* is the highly diffusible element. Now in the region of *θ* when the switch is activated the response *X*^∗^is expected to attain a value which is purely determined by local kinetics. Now noting that in this region, the reaction is essentially a zeroth order reaction, we expect that all the available X is essentially converted to *X*^∗^ and that practically no *X* remains. When taken together with the fact that *X* is essentially uniform, we see that this results in a situation where the X level everywhere is very low, with the profile being practically uniform, while in the region of switching, the *X*^∗^“absorbs” essentially all the available X in the domain. In this region, it is clear that *X*^∗^ is strongly switched on and in the limiting case, the response profile attains an essentially uniform level *X*_0_, where *X*_0_ is determined from the global conservation condition

(45) $$\begin{array}{l} X_{0}(\theta_{2}-\theta_{1})+X(2\Pi )=X_{\text{tot}}(2\Pi )\\ \;\;\;\;X_{0}=(X_{\text{tot}}-X)2\Pi /(\theta_{2}-\theta_{1}) \end{array}$$

Now since *X* is low, we find that in the limit *X*^∗^may approach a value close to *X*_*tot*_2*Π*/(*θ*_2_−*θ*_1_) and therefore can become very strongly amplified locally. We notice here why the response may become quite large, and indeed may even exceed *X*_*tot *_locally. Thus diffusion of one element combines to strongly enhance the switching effect.

#### Comparison with other monostable switches

The effect of diffusion of one component in switching discussed previously is quite striking. Since that is based on a particular, though frequently used, model of a switch, we briefly compare the effects of switching in other monostable switches. Suppose we had a simple switch arising through some Hill co-operativity for instance, and driving a response element. The model for such a switch would be

(46) $$\begin{array}{l} \;\text{dR}/\text{dt}=-k_{f}f(S)(R)+k_{r}R^{*}+k_{d1}\frac{\partial^{2}R}{\partial \theta^{2}}\\ dR^{*}/\text{dt}=k_{f}f(S)(R)-k_{r}R^{*}+k_{d2}\frac{\partial^{2}R^{*}}{\partial \theta^{2}} \end{array}$$

The switching effect is contained in the nonlinearity in the function f(S). In particular this function can approach a step function in certain cases. In the non-diffusible case, the behaviour of this module is easily understood. In the region where the signal is such that f(S) is essentially 1, a high response is expected, while in other regions, the response is practically 0.

We now examine this module in the case where a diffusible element is present. Suppose the response is the highly diffusible element, then it will attain a practically uniform steady state profile. On the other hand suppose R is the diffusible element. In this case, from the analysis of the previous section, we find that

(47) $$\begin{array}{l} R^{*}=R_{\text{tot}}\frac{k_{f}f(S)}{k_{f}<f(S)>+k_{r}} \end{array}$$

We see by inspecting this equation, that it is possible for the response to indeed exceed *R*_*tot*_. An example of this is when *k*_*f *_>* k*_*r *_the signal is switched in a narrow region, leading to a small value for the spatial average of *f*(*S*), which is completely dwarfed by the local value of f(S). Incidentally this exceeding of the locally available *R*_*tot*_ can be realized even for non switch-like upstream signals.

### Appendix F: Analytical results of the bistable switch model

We presented numerical results for the bistable switch in the text. These results can be explained analytically as follows.

First in the non-diffusible case, the system reaches a steady state governed by

(48) $$\begin{array}{r} k_{0}+k_{1}S(\theta )-k_{2}X^{*}-k_{21}X^{*}Y^{*}=0\\ \frac{-k_{3}X^{*}Y^{*}}{K_{M3}+Y^{*}}+\frac{k_{4}Y}{K_{M4}+Y}=0 \end{array}$$

where *Y *=* Y*_*tot*_−*Y*^∗^.

The behaviour of this system simply reflects the local bistability. Now in the case where *Y * (and *Y*^∗^) are highly diffusible, we see that *Y * and *Y*^∗^will attain a spatially homogeneous profile. The steady states for the full system are given by

(49) $$\begin{array}{r} \frac{-k_{3}<X^{*}>Y^{*}}{K_{M3}+Y^{*}}+\frac{k_{4}Y}{K_{M4}+Y}=0\\ k_{0}+k_{1}S(\theta )-k_{2}X^{*}-k_{21}X^{*}Y^{*}=0 \end{array}$$

The second equation may be written as

(50) $$\begin{array}{l} X^{*}=\frac{\left(k_{0}+k_{1}S(\theta )\right)}{k_{2}+k_{21}Y^{*}} \end{array}$$

This equation immediately reveals that the spatial heterogeneity of *X*^∗^directly arises from the signal rather than through any spatial switching effect. Note that in this equation *Y*^∗^ is homogeneous but an implicit function of *X*^∗^. Averaging both sides of this equation spatially, we obtain,

(51) $$\begin{array}{lcrr} <X^{*}> & = & \frac{\left(k_{0}+k_{1}<S(\theta )>\right)}{k_{2}+k_{21}Y^{*}} & \end{array}$$

This is coupled to the other equation relating *Y*^∗^to the spatial average of *X*^∗^. By rewriting the equation in terms of the spatial average of *X*^∗^ we find in this module that the spatial average of *X*^∗^(along with *Y*^∗^) satisfies the same algebraic equation as the temporal module, where the signal of the temporal module is replaced by the spatial average of the signal. This immediately indicates that if the spatial average of the signal is in the bistable regime then there are two distinct steady states for <*X*^∗^> and *Y*^∗^, one corresponding to the upper branch and one to the lower branch of the bifurcation diagram. Now using this in the above equation relating *X*^∗^ to the signal, and eliminating *Y*^∗^in terms of <*X*^∗^>, we see that there are two distinct steady states, each corresponding to spatial variations about the respective homogeneous steady states.

In summary, in the highly diffusible case, the response of the module results in multiple heterogeneous steady states but without any spatial switching.

### Appendix G: Transcritical switch model

Here, we examine a different kind of monostable switch, which arises from a positive feedback, which is distinct from the switches examined in the text. This is a positive feedback module which results in a switch with a transcritical bifurcation. We examine this module separately, as it is of interest to examine whether this distinct kind of switching behaviour yields results which are different from the other monostable switch and has been used in modelling [59,60]. This switch involves a simple positive feedback circuit where the active form of a species activates the active form of another species Y which feeds back to enhance the production of *X*^∗^. In this case, we will consider the external signal to regulate the degradation of *X*^∗^. Here *X*^∗^ is regarded as the output of the module.

#### The model equations

The equations governing the Transcritical Switch module are given by

(52) $$\begin{array}{l} \frac{\partial X^{*}}{\partial t}=-k_{\text{sx}}SX^{*}+k_{1}XY^{*}+D_{X}\frac{\partial^{2}X^{*}}{\partial \theta^{2}}\\ \frac{\partial Y^{*}}{\partial t}=k_{2}X^{*}Y-k_{-y}Y^{*}+D_{Y}\frac{\partial^{2}Y^{*}}{\partial \theta^{2}} \end{array}$$

In the above equations, Y is the intermediate feedback element. The signal S acts to degrade *X*^∗^ and *k*_*sx*_ denotes the associated rate constant. *k*_1_ is the kinetic rate constant associated with the positive feedback between the species *X* and *Y *. *k*_2_ denotes the rate constant for the conversion of *Y * to *Y*^∗^. *k*_−*y*_ is the rate constant for the constitutive conversion between the active and inactive forms of Y.

Now an analysis of the temporal dynamics of this circuit (discussed briefly later) clearly reveals the presence of a transcritical bifurcation. Thus, with regard to the effect of the signal *S*, the circuit behaves like a switch, which is different from both the monostable and bistable switches considered earlier. In particular, if the signal exceeds a particular threshold (the transcritical bifurcation point), the only stable response is the zero response. This is because for this range of signals, the positive feedback is not strong enough to sustain the production of a non-zero response in the face of the strong degradation (which is mediated by the external signal). We note in passing that if the signal were acting to increase the production of *X*^∗^ (rather than degrade it) then the transcritical bifurcation would be broken by the presence of the signal, though a switch-like effect would still be evident if the signal were at low levels. We adopt a representative circuit model where the transcritical bifurcation structure is preserved, and hence consider the signal degrading *X*^∗^.

#### Analysis of the transcritical switch model

The transcritical switch module is distinct from the monostable and bistable modules considered earlier. This is a module which also involves positive feedback, but where the switching (or more precisely thresholding) effect arises through a transcritical bifurcation. This module consists of two species X and Y, where the element Y is the intermediate element through which X catalyzes its own production. The signal (assumed non-zero) catalyzes the degradation of *X*^∗^. Since the signal catalyzes the backward reaction, this system exhibits a transcritical bifurcation, which is not destroyed by the presence of the signal.

We note from a basic analysis of the module, that two steady states exist (*X*^∗^ = 0) and a non-zero steady state value. While performing simulations for different homogeneous values of the signal, we find that above a critical value of the signal, the observed steady state is *X*^∗^ = 0. This is easily understood in terms of the characteristic transition associated with a transcritical bifurcation: there are two steady states which exchange stabilities when the critical level of the signal is crossed. Below the critical level, the positive feedback is the dominant effect and a non-zero steady state emerges as the observed state. When the signal crosses a threshold, the positive feedback is defeated by the degradation effect associated with the signal, resulting in a zero response.

**Figure 14:**
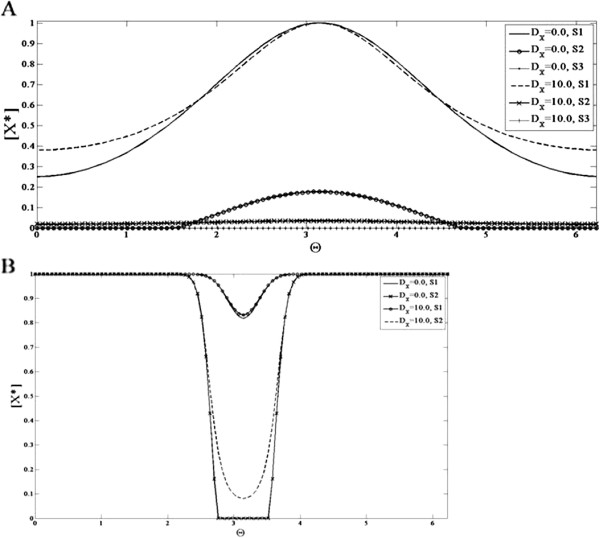
**Response of the transcritical bifurcation switch module.** The response of the module to different (**A**) gradient signals *S*1,*S*2 and *S*3 and (**B**) localized signals *S*1 and *S*2 for the non-diffusible and highly diffusible cases is shown. (**A**) Non-diffusible case: S1- is above the switch threshold (i.e. it is small enough for a non-zero steady state output: see text) in the entire domain (solid line), S2 is partially above and below the switching threshold (solid line with circles) and S3 is below the switch threshold in the entire region (solid line with dots at zero), Diffusible case: S1 (dashed line), S2 (solid line with x markers) and S3 (solid line with plus signs at zero). (**B**) Non-diffusible case: S1 is above the switch threshold in the entire domain (solid line), S2 is partially above and below the switching threshold (solid line with x markers), Diffusible case: S1 (solid line with circles) and S2 (dashed line). For the non-diffusible case, in the spatial regions where the signal value crosses the ”switch” threshold, the response exhibits a switch. This can be seen for S2 in (**A**) and in (**B**), where a part of the concentration profile shows non-zero spatial variation, and the other part is at zero or switched off. These plots clearly illustrate the presence of a spatial switch. Note that for S3 in (**A**) the profiles are at zero steady state in the entire domain. In the highly diffusible case, the spatial concentration profile of *X*^∗^has weaker spatial variation and the switching nature of the response is not observed here.

First we examine the case when none of the elements diffuse. If the input gradient signal is such that the local signal level at every location is completely below the critical value of the signal then the profile of *R*^∗^follows a spatial variation that is qualitatively opposite to that of the signal (note that the signal is acting to degrade the response, so this is expected). If the signal gradient is such that locally it is everywhere above the critical threshold, then a homogeneous zero response is observed (Figure 14). Both these results are easily understood in terms of the dynamics of the module. However, if the gradient is above the critical value for some regions in space, then for those regions in space the concentration of *R*^∗^ is zero and for the regions where the signal is below the threshold, it is non-zero and follows a variation opposite to the signal. This result illustrates the switching effect in spatial gradients. Similar observations hold for a localized signal input (Figure 14).

Finally, we examine the case where highly diffusible elements are present in the network. If the response *X*^∗^ is diffusible then it naturally exhibits no spatial variation. We consider a situation where Y is highly diffusible, If the signal is everywhere below the critical value corresponding to the bifurcation, the steady state concentration profile is qualitatively opposite to that of the signal and is more spread out when compared to the case when none of the elements diffuse. When some or all of the local signal crosses beyond the critical value of *S*, the profile of *X*^∗^ has very weak spatial variation. Moreover the switch-like behaviour seen in the spatial profile in the case when none of the elements diffuse is absent here and the profile does not reach a zero value (Figure 14). When a localized signal input is applied, the profile of *X*^∗^is smoothly localized. For signals with amplitudes below and above the critical value, *X*^∗^ always remains non-zero- unlike the previous case for the same signals (Figure 14). Thus there is no manifestation of the switch-like behaviour in the spatial profile. If a signal is everywhere above a certain threshold (see below) then a spatially uniform zero response is observed, even with a gradient input.

It is worth pointing out that the diffusible element Y has the effect of preserving the switch-like behaviour in the steady state response to homogeneous signals, but negates the effect of this switch in spatial signal transduction in response to gradient signals.

These results may be explained analytically as follows. We first consider the non-diffusible case. The steady states of the module are obtained from the following equations.

(53) $$\begin{array}{c} X^{*}=X_{\text{tot}}\frac{k_{1}Y^{*}}{k_{1}Y^{*}+k_{\text{sx}}S}\\ Y^{*}=Y_{\text{tot}}\frac{k_{2}X^{*}}{k_{2}X^{*}+k_{-y}} \end{array}$$

It is obvious from above that one steady state corresponds to *X*^∗^ =* Y*^∗^ = 0. A second non-zero steady state is also easily obtained. We analyze the above module in the case where the Y reaction is far from saturation (the saturation effect in X is however retained to preserve the transcritical bifurcation). In this case we have

(54) $$\begin{array}{l} X^{*}=X_{\text{tot}}\frac{k_{1}Y^{*}}{k_{\text{sx}}S+k_{1}Y^{*}}\\ Y^{*}=Y_{\text{tot}}\frac{k_{2}X^{*}}{k_{-y}} \end{array}$$

This results in two steady states which are

(55) $$\begin{array}{l} X^{*}=0\\ X^{*}=X_{\text{tot}}-\frac{k_{\text{sx}}Sk_{y}}{k_{1}Y_{\text{tot}}k_{2}} \end{array}$$

Thus these simplified equations capture the switch behaviour and the transcritical bifurcation of the full module. The behaviour of the response *X*^∗^to spatially graded signals is understood in terms of the transcritical bifurcation and hence exhibits a spatial switching behaviour.

Now we examine the effect of a highly diffusible Y. Here the steady state equations (assuming the Y reaction is far from saturation) is given by

(56) $$\begin{array}{l} X^{*}=X_{\text{tot}}\frac{k_{1}Y^{*}}{k_{\text{sx}}S+k_{1}Y^{*}}\\ Y^{*}=Y_{\text{tot}}\frac{k_{2}<X^{*}>}{k_{-y}} \end{array}$$

This results in the steady state for *X*^∗^as

(57) $$\begin{array}{l} X^{*}=X_{\text{tot}}\frac{A<X^{*}>}{k_{\text{sx}}S+A<X^{*}>} \end{array}$$

where *A *=* Y*_*tot*_*k*_1_*k*_2_/*k*_−*y*_. A careful examination of the above steady state equation indicates firstly that there is a homogeneous zero steady state and also that if the response is not zero everywhere then it equals the right hand side of the equation, where the only spatial variation is through the signal in the denominator. This indicates that this steady state solution will reflect a spatial variation which is directly driven and in some sense commensurate with the external signal with no spatial switching effect.

It is worth examining whether a uniform zero steady state can appear in a spatially graded signal, since such a response can be obtained in a homogeneous signal of sufficiently high magnitude. By averaging both sides of the equation, and cancelling out <*X*^∗^> assuming it is non-zero, we find that the response has to satisfy the equation

(58) $$\begin{array}{l} \frac{1}{2\Pi }\int_{0}^{2\Pi }X_{\text{tot}}\frac{k_{1}A}{k_{\text{sx}}S(\theta )+k_{1}A<X^{*}>}=1 \end{array}$$

We see here that if S is of sufficiently high magnitude everywhere, this equation cannot be satisfied for a positive concentration profile, and thus we may expect a spatially uniform zero response under these conditions (incidentally for high signal, the assumption of Y being far from saturation continues to hold). This is indeed what is observed: when the module is subject to a gradient signal of sufficiently high mean value, the steady state attained is the spatially uniform zero steady state. This example showcases how the effect of the transcritical bifurcation is present in spatial signalling, with a highly diffusive intermediate element: no spatial switch is observed in a gradient signal. However the effect is seen in the nature of the response profile, when one considers a sequence of inputs of increasing mean values.

## Competing interests

Both Authors’ declare that they have no competing interest.

## Authors’ contributions

Both authors AA-N and JK planned the work, both authors AA-N and JK directly contributed to the results presented here and both authors AA-N and JK were involved in the writing of the manuscript. All authors read and approved the final manuscript.

## Supplementary Material

Additional file 1 Additional file contains supplementary information about the parameters in all figures. Click here for file
