# Supplementary material for: An investigation of spatial signal transduction in cellular networks
Source: BMC Syst Biol. 2012 Jul 5;6:83. doi: 10.1186/1752-0509-6-83 (PMC3537682; doi:10.1186/1752-0509-6-83)
Supplement: Additional file 1 — Additional file contains supplementary information about the parameters in all figures. [file 1752-0509-6-83-S1.pdf]

Supplementary information

In this section, the signal and parameter values in each module are outlined. In all the simulations, unless otherwise mentioned, the initial conditions used are steady state values for a basal signal.

Figure 2 - Parameters for the Coherent Feedforward module

(A) $S = 1 + 0.3cos\theta$ and (B) $S = A * exp((x - x_o)/\alpha)$ where $A = 1.0$ and $\alpha = 0.1$, here and everywhere below, unless otherwise mentioned, $x_o = \pi$ (centre of domain) Parameter values: $k_{sx} = 1.0, k_{sy} = 1.0, k_x = 0.0, k_{-x} = 1.0, D_X = 0.0, k_{xr} = 10.0, k_y = 0.0, k_{-y} = 1.0, k_{yr} = 1.0, D_Y = 0.0, k_r = 0.0, k_{-r} = 1.0, X_{tot} = 1.0, Y_{tot} = 1.0, R_{tot} = 1.0$. $D_X = 10.0$ and $D_Y = 10.0$ for the highly diffusible cases. In this module k_{xr} and k_{yr} were varied to modulate the strength of the associated pathways.

Figure 3 - Parameters for the Incoherent Feedforward module

A: $S = 1+0.3cos\theta$; B:$S = a+A*exp((x-x_o)/\alpha)$ where $A = 1.0$, $a = 0.1$ and $\alpha = 0.1$. The parameter values are: $k_{sx} = 1.0; k_{-x} = 2.0; k_{xr} = 1.0; k_{sy} = 1.0; k_{-y} = 1.0; k_{yr} = 1.0;$ $k_x = 0.0; D_X = 0.0; k_y = 0.0; D_Y = 0.0; k_r = 0.0; k_{-r} = 0.0; X_{tot} = 1.0; Y_{tot} = 1.0; R_{tot} = 1.0$. $D_X = 10.0$ and $D_Y = 10.0$ for the highly diffusible cases. C: The parameter values are: $k_{sy} = 2.0; k_{-y} = 0.5; k_{xr} = 1.0; k_{sx} = 1.0; k_{-x} = 0.0; k_{yr} = 1.0;$ $k_y = 0.0; D_Y = 0.0; k_x = 1.0; D_X = 0.0; k_r = 0.0; k_{-r} = 1.0; X_{total} = 1.0; Y_{total} = 1.0; R_{total} = 10.0$.

Figure 4 - Parameters for the Positive Feedback module

A: $S = 0.5+0.3cos\theta$; B: $S = a+A*exp((x-x_o)/\alpha)$ where $A = 1.0$, $a = 0.1$ and $\alpha = 0.1$. The parameters are $k_{sx} = 1.0, k_x = 0.0, k_{-x} = 1.0, D_X = 0.0, k_{ry} = 1.0, k_y = 0.0, k_{-y} = 1.0, D_Y = 0.0, k_{xr} = 1.0, k_r = 0.0, k_{-r} = 1.0, D_r = 0.0; X_{tot} = 1.0; Y_{tot} = 1.0; R_{tot} = 1.0$. $D_Y = 10.0$ for the highly diffusible case. k_{yx} is varied here to modulate the effect of the feedback strength.

Figure 5 - Parameters for the Negative Feedback module

A: $S = 0.5+0.3cos\theta$; B: $S = A*exp((x-x_o)/\alpha)$ where $A = 1.0$ and $\alpha = 0.1$. The parameters for this module are: $k_{sx} = 1.0, k_x = 0.0, k_{-x} = 1.0, D_X = 0.0, k_{yr} = 5.0, k_y = 0.0, k_{-y} = 1.0, D_Y = 0.0, k_{xr} = 1.0, k_r = 0.0, k_{-r} = 1.0, D_R = 0.0; X_{tot} = 1.0; Y_{tot} = 1.0; R_{tot} = 1.0$. $D_Y = 10.0$ for the highly diffusible case. k_{yx} is varied here to modulate the effect of the feedback strength.

Figure 6 - Parameters for the Cyclic Reaction network module

A case with n=4 species in the module was analysed. A. $S = 1 + 0.3cos\theta$ and B. $S = A * exp((x - x_o)/\alpha)$ (where $A = 1.0, a = 0.1$ and $\alpha = 0.1$) The parameters for this module are: $k_s = k_2 = k_3 = k_4 = 1.0, D_{X1} = D_{X2} = D_{X3} = D_{X4} = 0.0$.

Figure 7 - The Cyclic Reaction network module with highly diffusible elements

$D_{X1} = 10.0$, $D_{X3} = 10.0$, when each of those elements was made highly diffusible. The parameters and the signal input are the same as outlined in Fig. 6.

Figure 8 - Parameters for the Monostable Switch Module

(A,C). $S = 1+0.3sin\Theta$ and (B,D). S is a square pulse: $S_{basal} = 0.1$ and $S_{maximum} = 5.0$. The parameters for this module are: $k_{sx} = 0.5, U = 0.5, K_{M1} = 0.01, K_{M2} = 0.01, D_X = 0.0, D_{X^*} = 0.0, [X] + [X^*](initially) = 0.2$. $D_X = 10.0$ when considering the diffusible case.

Figure 9 - Parameters for the Mutual Inhibition Bistable switch module

Note that in the analysis of this module different initial conditions have been used for the cases analysed and are outlined below. The effect of weak diffusion was also examined in this module. A. $S = 1 + 0.3cos\Theta$, B. $S = 1.6 + 0.3cos\Theta$. (C,D). $S = a + A * exp((x - x_o)/\alpha)$, where $a = 1.69$, (C) $A = 0.05$ and (D) $A = 0.1$. The parameters for this module are: $k_o = 0.0, k_1 = 0.05, k_2 = 0.1, k_{21} = 0.5, D_X = 0.0, k_3 = 0.2, K_{M3} = 0.05, k_4 = 1.0, K_{M4} = 0.05, D_Y = 0.0$. $X_{initial} = 0.1554$ and $Y_{initial} = 0.8617$ (E) For the weakly diffusible case $S = 1.27 + 0.3cos\Theta$ and $D_Y = 0.01$. $X^*_{initial} = 0.5632$ and $Y^*_{initial} = 0.02551$ at $\theta = 0$ to 0.46π and 1.52π to 2π. $X_{initial} = 0.111$ and $Y_{initial} = 0.9443$ at $\theta = \pi/2$ to $3\pi/2$. $Y_{tot} = 1$ everywhere.

Figure 10 - Parameters for the Mutual Inhibition Bistable switch module with highly diffusible elements

D_Y=10.0 and the other parameters and the signal input are the same as outlined in Fig. 9. Note that in (E) $S = 1.3 + 0.3cos\Theta$ and $X^*_{initial} = 0.8855$ and $Y^*_{initial} = 0.01361$.

Figure 11 - Parameters for the Negative Feedback Oscillator module

Note that in this module, a case with a weakly diffusible species was also analysed. (A,B,C) $S = 0.3+0.2cos\theta$ (D) $S = A * exp((x - x_o)/\alpha)$, where $A = 4.0$. The parameters for this module are: $k_o = 0.0, k_1 = 1.0, k_2 = 0.01, k_{21} = 10.0, D_X = 0.0, k_3 = 0.1, k_4 = 0.2, k_5 = 0.1, k_6 = 0.05, K_{M3} = K_{M4} = K_{M5} = K_{M6} = 0.01, D_Y = 0.0$. (E,F) For the weakly diffusible case $S = 0.3 + 0.2cos\theta$ and $D_Y = 0.5$.

Figure 12 - Parameters for the Negative Feedback Oscillator module with highly diffusible elements

D_Y=10.0 and the other parameters and the input signals are the same as in Fig. 11.

Figure 13 - Parameters for the Transcritical Bifurcation module

The parameters for this module are: $k_{sx} = 1.0, k_1 = 1.0, D_{X^*} = 0.0, k_2 = 1.0, k_{-y} = 1.0, D_{Y^*} = 0.0, X_{tot} = 1.0; Y_{tot} = 1.0$. A. $S1 = 0.3 + 0.3 cos\theta$, $S2 = 1.0 + 0.3 cos\theta$ and $S3 = 2.0 + 0.3 cos\theta$. B. $S = A * exp((x - x_o)/\alpha)$ where $\alpha = 0.1$, $S1$: $A = 0.1$ and $S2$: $A = 5.0$.
